# Ancient DNA from the Upper Paleolithic mammoth ivory of Hohle Fels, Germany

**DOI:** 10.1038/s41598-026-46761-x

**Published:** 2026-05-14

**Authors:** Kelsey N. Moreland, Sibylle Wolf, Dorothée G. Drucker, Arianna Weingarten, Ella Reiter, Maria A. Spyrou, Nicholas J. Conard, Cosimo Posth

**Affiliations:** 1 Archaeo- and Palaeogenetics, Institute for Archaeological Sciences, Department of Geosciences, University of Tübingen, Tübingen, Germany; 2 Centre for Palaeogenetics, Stockholm, Sweden; 3 Senckenberg Centre for Human Evolution and Palaeoenvironment, University of Tübingen, Tübingen, Germany; 4 Early Prehistory and Quaternary Ecology, University of Tübingen, Tübingen, Germany; 5 Biogeology, Department of Geosciences, University of Tübingen, Tübingen, Germany; 6 HUMAN ORIGINS—Cluster of Excellence for Integrative Human Origins Studies (EXC 3101), University of Tübingen, Tübingen, Germany

**Keywords:** Evolution, Genetics

## Abstract

Hohle Fels, a cave in southwestern Germany, hosts one of the richest and best-preserved Upper Paleolithic ivory assemblages, with some of the earliest examples of figurative art, musical instruments, and personal ornamentation. Despite this, its ivory had never been tested for ancient DNA (aDNA). Here, we genetically analyze 25 anthropogenic ivory fragments from Upper Paleolithic archaeological layers and demonstrate the feasibility of aDNA recovery from non-permafrost ivory. We show that cementum yields significantly more and higher-quality aDNA than dentin, making it the preferred ivory material for paleogenetic investigations. Low-coverage genome-wide data revealed a female bias, potentially reflecting either natural herd structure or biases in the procurement of ivory material. Mitochondrial DNA enrichment allowed the reconstruction of twelve complete mtDNAs, revealing that the specimens were sourced from multiple distinct mammoth lineages. Together, these results demonstrate that archaeological ivory can provide unprecedented insights into both mammoth biology and Upper Paleolithic human behavior, including hunting strategies, raw material selection, and symbolic practices of European hunter-gatherers.

**Supplementary Information:**

The online version contains supplementary material available at 10.1038/s41598-026-46761-x.

## Introduction

Ivory and the animals it comes from have long symbolized beauty, rarity, and strength in human societies. Since the Upper Paleolithic (ca. 45,000 to 11,700 years cal. BP), woolly mammoth ivory was extensively used by humans to craft personal ornaments, tools, and musical instruments across the northern hemisphere^1–5^. Although Upper Paleolithic mammoth ivory has been extensively studied^6–12^, many aspects of the human-mammoth interactions during this period remain unclear, including whether humans selectively targeted specific sexes or herds, how ivory was transported or exchanged across landscapes, and why the use of ivory varied between cultural periods such as the Aurignacian, Gravettian, and Magdalenian. Understanding the biology and population structure of exploited mammoths is essential for interpreting patterns of ivory selection and use in prehistoric cultures.

Despite substantial advances in mammoth genomics, our understanding of European populations associated with Upper Paleolithic humans remains incompletely understood. This is largely due to uneven preservation, as European remains are non-permafrost, leading to lower success rates and lower-quality DNA^13,14^. Nevertheless, maternally inherited mitochondrial genomes (mtDNA) recovered from European woolly mammoths indicate a shift from mtDNA Clade III to mtDNA Clade I between ~ 24.5 and 16.2 thousand years ago (ka)^15–17^, suggesting a population turnover around the time of the Last Glacial Maximum and implying that Magdalenian mammoths belonged to a different population than those exploited during the Aurignacian and Gravettian. Low-coverage nuclear DNA is also realistically accessible and allows for sex determination. Using this approach, a male-biased sex ratio was found in naturally accumulated Siberian mammoths^18^, and a study from the Eastern European kill site Kostenki 11-Ia found a female-biased ratio in anthropogenically mass accumulated mammoths^19^, more closely reflecting herd structures. Additional specimens from the Upper Paleolithic are needed to provide finer-scale resolution and stronger connections to archaeological questions, thereby avoiding over-extrapolation from distant populations.

One promising avenue to expand the sample pool for European mammoth genetics is the study of archaeological mammoth ivory in museum and excavation collections. Although these artifacts have long attracted archaeological attention^4^, their potential as a source of ancient DNA remained unexplored. Additionally, because ivory reflects deliberate human selection, transport, and modification of mammoth tusks, its genetic analysis can provide unique insights into prehistoric human behavior different from that of bones or mass kill sites and completely inaccessible using naturally accumulated remains. For example, they may reflect cultural preferences for raw materials, possible sex-based selection of mammoth individuals for specific artifacts, and even long-distance exchange or mobility patterns^11,20,21^. Differences in how mammoth ivory was sourced and used across time and archaeological cultures, such as the Aurignacian, Gravettian, and Magdalenian, may provide insights into changing subsistence strategies, symbolic practices, and human-animal relationships during the Upper Paleolithic.

To leverage the genetic potential of ivory, it is also essential to understand how the biological composition of mammoth tusks, which consist of dentin and cementum, affects DNA preservation. Modern elephant tusk DNA acquisition guidelines assume the cementum layer, which forms the outermost part of the tusk and protects the inner dentin, harbors higher concentrations of preserved DNA^22,23^ based on parallels drawn from results on human teeth^24–27^. However, the differential preservation of aDNA within these components remains untested in both recently deposited and archaeological ivory. Developing an informed sampling strategy is critical for minimizing destructive analysis of these culturally and scientifically valuable objects while maximizing the recovery of genetic material.

The archaeological record from Hohle Fels cave in the Ach Valley of southwestern Germany provides an appealing framework to investigate these questions. Hohle Fels is a well-stratified site with evidence of extended hominin occupations since the Middle Paleolithic. It is particularly well known for its rich mammoth ivory assemblage, which includes some of the world’s oldest known musical instruments and figurative art^28–31^. Numerous animal remains were recovered from Aurignacian (~ 42 − 35 ka) and Gravettian (~ 35 − 30 ka) layers. The human groups exploited a wide range of species, including wild horse, reindeer, various cervids, mammoth, and small game^32,33^. Cut marks on mammoth bones indicate that proboscidean remains were processed at Hohle Fels during both the Aurignacian and the Gravettian. Alongside these iconic finds, abundant ivory byproducts from active working offer a robust cultural and chronological framework to investigate both technological use and genetic content of ivory. In this study, we target 25 mammoth ivory detritus byproducts of anthropogenic origin from Hohle Fels, sourcing from Upper Paleolithic layers, to (1) optimize sampling strategies for aDNA extraction from ivory, (2) verify the age of Magdalenian ivory flakes to confirm their stratigraphic assignment, (3) determine the sex of the mammoths exploited by humans at Hohle Fels, and (4) place the Hohle Fels ivory within the known matrilineal diversity of European mammoths, using these patterns to infer aspects of human selection, herd exploitation, and material use prior to and following the Last Glacial Maximum.

## Results

### Shotgun and capture sequencing

The 25 anthropogenic mammoth ivory pieces sampled in this study were selected across five major archaeological horizons from the Aurignacian, Gravettian and Magdalenian (Layers I–V), encompassing 14 sub-layers at Hohle Fels (Table 1; see Supplementary Materials and Figure S1 for more information). Recovered endogenous DNA from shotgun sequencing ranged from 0.005% to 20.199% (Supplementary Table S3) and showed expected damage patterns for partial USER treatment libraries (Figure S2). Despite sampling from a range of archaeological horizons, no significant difference was observed in aDNA preservation between archaeological layers or horizons (Figure S3), but one specific layer (AH Vb) showed heightened potential for aDNA recovery from its ivory specimens (Supplementary Materials).

**Table 1:** Sample information for the 29 ivory samples from 25 specimens analyzed in this study. Mitogenome completeness is calculated as breadth of coverage for the reconstructed consensus mtDNA sequences. Genetic sex is provided where available. Endogenous DNA is provided for unenriched shotgun sequencing mapped to the LoxAfr4 nuclear genome. Enrichment factor shows the fold increase in endogenous DNA mapping to the mammoth mtDNA reference genome after mtDNA targeted enrichment. Calculation details are provided in the Methods section. Timings for the archaeological contexts at Hohle Fels are as follows: Aurignacian (42 − 35 ka), Gravettian (35 − 30 ka), Magdalenian (17 − 14 ka). *Direct radiocarbon dates place these remains to a timing consistent with the Gravettian.

Lab ID	Archaeological Context	Material	Mitogenome Completeness (%)	Mean Mitogenome Coverage	Genetic Sex	Endogenous DNA (%)	Enrichment Factor (x increase)
HOLF001.A	Layer I, Magdalenian*	Dentin	96.46	19.53	M	0.135	1165.1
HOLF002.A	Layer I, Magdalenian	Dentin	43.08	2.50	-	0.017	451.5
HOLF003.A	Layer Vab, Aurignacian	Dentin	97.70	28.09	-	0.042	627.3
HOLF004.A	Layer IIa, Magdalenian*	Dentin	96.94	18.63	-	0.032	632.4
HOLF005.A	Layer IIb, Gravettian	Dentin	3.24	0.56	-	0.012	undefined
HOLF006.A	Layer IIb, Gravettian	Dentin	90.98	9.64	-	0.023	560.2
HOLF007.A	Layer IIc, Gravettian	Dentin	95.60	11.82	F	0.13	843.8
HOLF008.A	Layer IIc, Gravettian	Dentin	3.16	0.52	-	0.005	undefined
HOLF009.A	Layer IIcf, Gravettian	Dentin	98.17	33.64	M	0.185	665.0
HOLF010.A	Layer IIcf, Gravettian	Dentin	0.00	0.00	-	0.008	undefined
HOLF010.B	Layer IIcf, Gravettian	Cementum	98.16	36.29	F	1.455	452.6
HOLF011.A	Layer IIdb, Aurignacian	Dentin	0.00	0.08	-	0.007	28.8
HOLF012.A	Layer IId, Aurignacian	Dentin	90.90	9.00	F	0.095	771.4
HOLF013.A	Layer IIIa, Aurignacian	Dentin	6.34	0.81	-	0.016	65.0
HOLF014.A	Layer IIIa, Aurignacian	Dentin	0.21	0.26	-	0.006	undefined
HOLF015.A	Layer IIIb, Aurignacian	Dentin	0.56	0.19	-	0.013	undefined
HOLF016.A	Layer IIIb, Aurignacian	Dentin	77.12	6.42	-	0.05	570.9
HOLF017.A	Layer IV, Aurignacian	Dentin	28.88	1.87	-	0.059	undefined
HOLF018.A	Layer IV, Aurignacian	Dentin	0.09	0.14	-	0.009	59.3
HOLF019.A	Layer Va, Aurignacian	Dentin	0.00	0.07	-	0.007	undefined
HOLF020.A	Layer Va, Aurignacian	Dentin	2.75	0.57	-	0.015	undefined
HOLF021.A	Layer Vb, Aurignacian	Dentin	95.18	13.70	-	0.042	997.3
HOLF022.A	Layer Vb, Aurignacian	Dentin	98.92	123.06	M	20.199	250.8
HOLF023.A	Layer IIc, Gravettian	Dentin	64.31	3.83	-	0.02	undefined
HOLF023.B	Layer IIc, Gravettian	Cementum	14.31	1.14	-	0.023	undefined
HOLF024.A	Layer IV, Aurignacian	Dentin	16.42	1.20	-	0.012	506.5
HOLF024.B	Layer IV, Aurignacian	Cementum	96.25	28.08	F	1.561	317.5
HOLF025.A	Layer Vaa, Aurignacian	Dentin	22.45	1.56	-	0.008	526.0
HOLF025.B	Layer Vaa, Aurignacian	Cementum	96.33	16.19	F	0.155	280.3

MtDNA in-solution capture was effective in increasing the recovery of mtDNA from the pieces compared to the shotgun sequencing results (Supplementary Tables S4, S5), with a 514x average increase of mtDNA percentage (*p* < 0.03; Supplementary Table S1). Twelve mitogenomes were reconstructed to > 90% completeness (Table 1), representing 48% of the sampled specimens.

### Radiocarbon dating

Three ivory pieces with reconstructed mitogenomes were directly dated at ETH Zurich. Only two of the three Magdalenian associated pieces had successful aDNA recovery. These two pieces were dated to the Gravettian. One Aurignacian piece was also dated and fit to its archaeological context (Supplementary Table S1, S10; Figures S4, S5). No specimens directly dated to the Magdalenian occupation of Hohle Fels were identified. We therefore did not have any mammoth individuals contemporaneous with the Hohle Fels Magdalenian occupation in our analyses, raising questions about the existence and use of mammoths in the Magdalenian of southwest Germany in general.

### Comparison of cementum and dentin

**Fig. 1:**
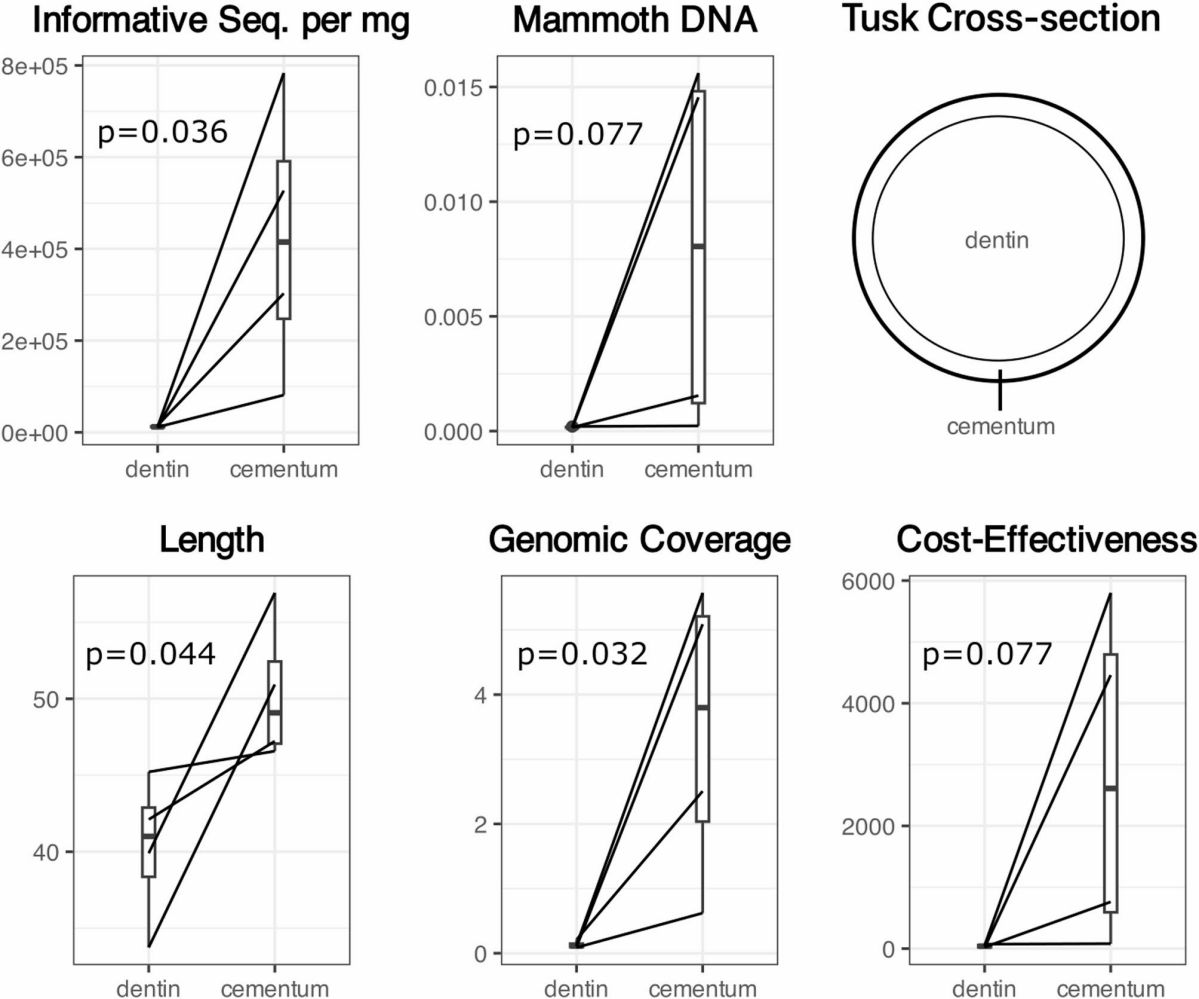
Comparison metrics for dentin and cementum components of the same anthropogenic mammoth ivory piece. P-values show over half of the metrics to be statistically significant despite the low available test samples. From left to right, the metrics displayed are the number of informative sequences in a 40 uL library normalized by sample weight, proportion of mammoth DNA in a library, average length of mapped reads in base pairs, total estimated genomic coverage obtainable per 50 mg of sample extracted, and cost-effectiveness, which is calculated as the number of unique base pairs gained from one million reads of sequencing effort per milligram of sample input. Precise calculation details for these metrics are provided in the Methods section.

Cementum and dentin from four ivory pieces were compared across five metrics (see Methods) using shotgun sequencing data. On average, cementum recovered 47.2× more endogenous mammoth DNA than dentin, although this difference was not statistically significant (*p* = 0.077; Fig. 1b). Cementum produced significantly more informative sequences per mg than dentin (34×; *p* = 0.036; Fig. 1a), indicating higher recovery efficiency even with the small sample size (*n* = 8, 4 pairs). DNA damage patterns were also slightly elevated in cementum compared to dentin, though not significantly, in contrast with previous findings in human teeth (Figure S6)^34^.

Although the quantity of yielded molecules is important, many analyses require confident variant calling and genome reconstruction, so genome complexity within the library is highly valuable. The libraries produced in this study were not sequenced to exhaustion, so instead the total genomic coverage (fold) in a library was estimated based on calculations from Parker et al^34^. and then normalized for milligrams sampled. Estimated genomic coverage normalized per mg was 26.3× higher in cementum than dentin (*p* = 0.032; Fig. 1d; Table 1). Cementum-derived reads were on average 10 bp longer (*p* = 0.044; Fig. 1c), and cost-effectiveness was 56× higher than dentin, though not statistically significant (*p* = 0.077; Fig. 1e). Collectively, these results show that cementum consistently outperformed dentin across multiple recovery metrics.

### Genetic sexing

**Fig. 2:**
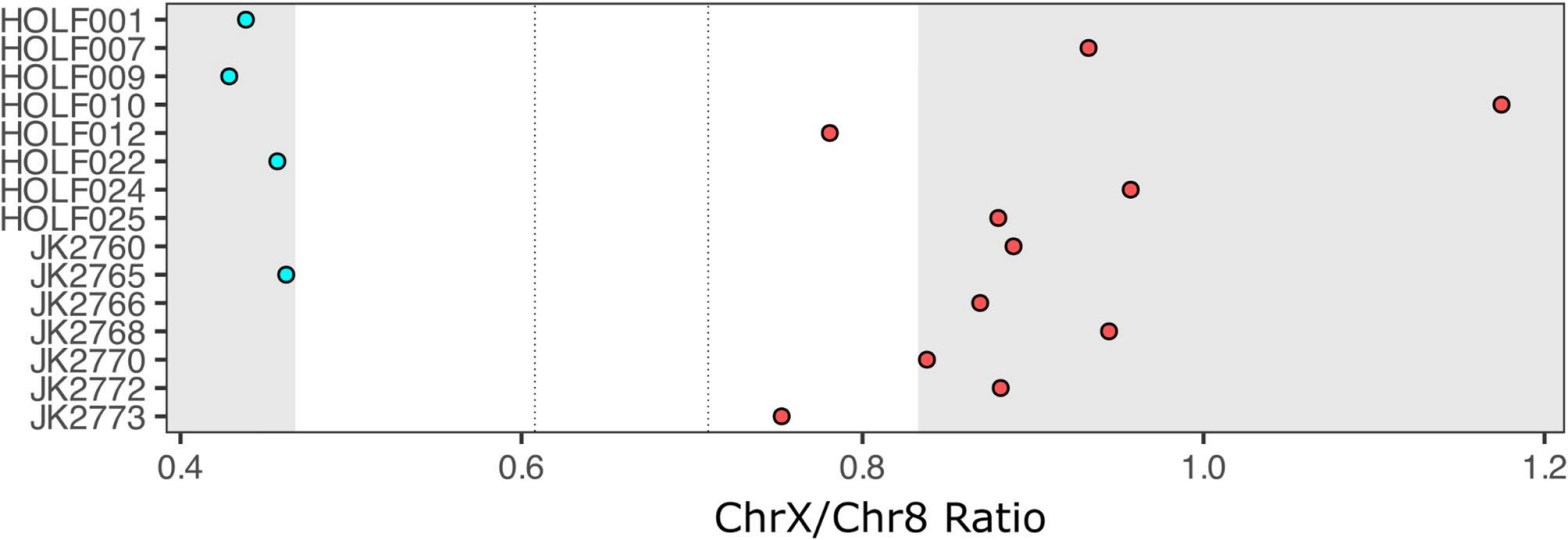
Sexing analysis where blue indicates mammoths assigned male and red indicates mammoths assigned female. HOLF specimens are newly sequenced in this study, and JK specimens are reanalyzed from previously published data^17^. Gray zones indicate unambiguous sex assignments, and dotted lines indicate confidence intervals based on training on the larger dataset by Pečnerová et al^18^. which were used to assign HOLF012 and JK2773 as females.

Of the 25 sequenced ivory pieces, eight had more than 100 reads mapping to the African savannah elephant X and 8 chromosomes from shotgun sequencing reads, allowing for genetic sexing following the method and categorization cutoffs outlined by Pečnerová et al.^18^ (Supplementary Table S6). The resulting sex ratio for Hohle Fels ivory specimens was 3:5 males to females with no significant trend by period (*p* = 1). In addition, aDNA from seven previously published Hohle Fels bone specimens from the Aurignacian and Gravettian in Fellows Yates et al.^17^ were re-mapped and assigned sexes for the first time (1:6 males to females; Supplementary Tables S6, S7). The resulting sex ratio for all sequenced Hohle Fels archaeological mammoth remains was 4:11 males to females (Fig. 2), demonstrating a female leaning signal. Though found to be not significant at the current sample size (*p* = 0.12), it is notably different from the male-biased ratio found in naturally accumulated mammoth remains (*p* = 0.0027)^18^. No temporal signal was able to be detected in this combined dataset due to low sample sizes when further divided by archeological culture (*p* = 0.57). Additionally, while in Hohle Fels the frequency of ivory obtained from female individuals is lower than in bones, the difference between the two ratios is not significant (*p* = 0.57), and neither material was significantly different from an even male/female individual sourcing (*p* = 0.73 and *p* = 0.13 for ivory and bone, respectively). Additional samples from Hohle Fels and other nearby caves across the same timespan are needed to increase the statistical power of these tests and to enable more robust evaluation of the observed sex-bias patterns.

### Phylogenetic placement

**Fig. 3:**
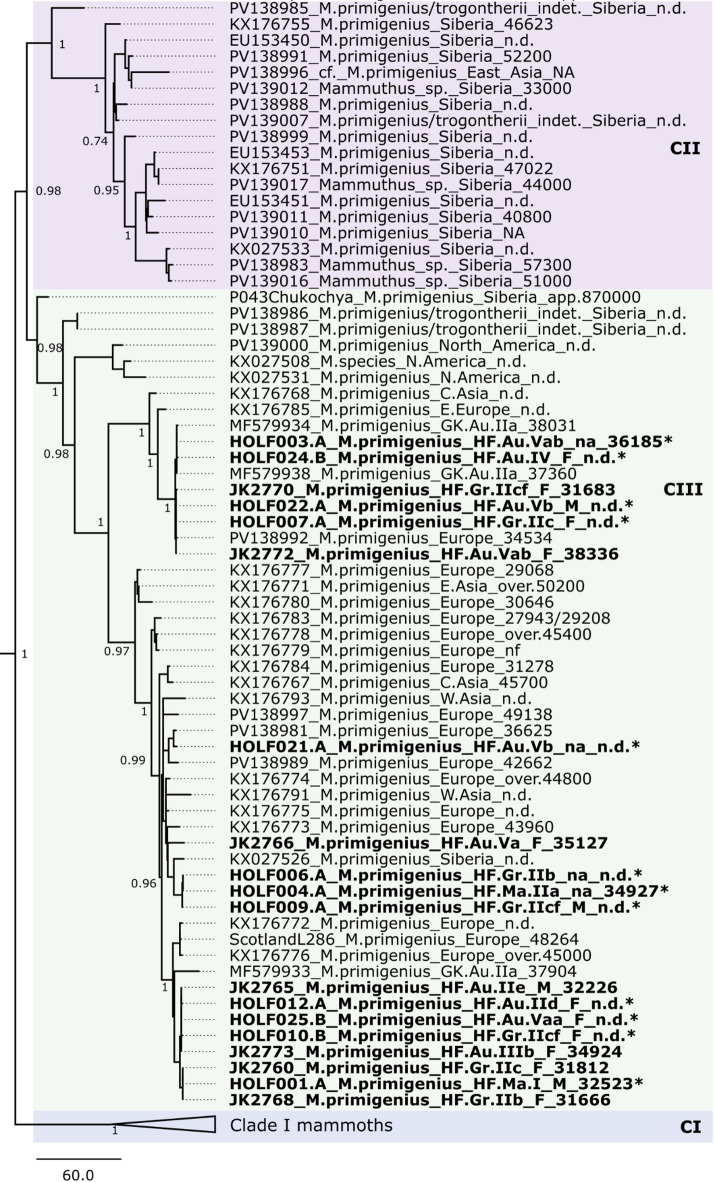
Phylogenetic reconstruction of woolly mammoth mtDNA diversity computed with Maximum Parsimony, partial deletion 80% and 1000 bootstrap iterations (bootstrap values reported on major nodes). Bolded individuals are from Hohle Fels cave and the twelve mitochondrial genomes newly reconstructed in this study are indicated with an asterix. All sequenced Hohle Fels mammoth mtDNAs group within the previously described Clade III mtDNA diversity. Non-dated samples are indicated with “n.d.” at the end of their labels.

The twelve newly generated mitogenomes above 90% complete were aligned with previously published mammoth mitogenomes (> 90% completeness; *n* = 217) and the Asian elephant mitogenome as an outgroup using MUSCLE^35^ (Supplementary Table S9). An 80% deletion maximum parsimony tree was built using MEGA^36^. All of the newly generated mitogenomes fall into Clade III as expected for European woolly mammoths before the Last Glacial Maximum, aligning with previous findings^15,16^^,17,^^37^. The mitogenomes reconstructed in this study doubles the complete Clade III mitogenomes from European woolly mammoths and increases the total complete Clade III mitogenomes by roughly 25%. The successful reconstruction of twelve mitogenomes in this study has also tripled the complete mitogenomes from the cave site, enabling us to now observe a mtDNA genetic diversity in a single cave that encompasses the entire variation of Clade III of Eurasian mammoths known so far (Fig. 3).

## Discussion

### Superior aDNA preservation in ivory cementum

Cementum material performed better than its respective dentin counterpart in all five of our calculated metrics, with significant differences detected in three out of the five despite the low sample size available for this comparison. For all three paired dentin-cementum specimens that yielded complete mitogenomes, success was driven by the cementum rather than the dentin, underscoring the critical impact of sampling strategy on downstream analyses. This outcome aligns with expectations based on studies on human teeth, where cementum has been generally assumed to preserve DNA better than dentin^22^^,23^ for both mtDNA^24^^,25^ and nuclear DNA^26^^,27^^,34^. However, this is the first direct demonstration on ivory, which differs chemically and structurally from teeth^38^^,39^. Our results therefore confirm that, as in teeth, ivory cementum provides superior aDNA preservation and should be prioritized for targeted sampling in archaeological contexts, while also minimizing unnecessary damage to valuable artifacts. These results are likely also applicable to non-anthropogenic mammoth tusk material as well as ancient or modern elephant tusk and ivory materials. One specimen, HOLF023, had poor aDNA recovery in both dentin and cementum, underscoring that individual preservation conditions impact aDNA recovery from cementum as well.

As cementum constitutes only a small fraction of tusk volume, its availability in archaeological assemblages is an important consideration. Though modern ivory carvings are typically fashioned from dentin due to its volume and structural properties^40^ evidence from Hohle Fels and Vogelherd, another cave site in the Swabian Jura, suggests a different pattern in the Upper Paleolithic. In both Gravettian and especially Aurignacian contexts, cementum appears to have been used deliberately, sometimes serving as a functional or aesthetic element of artifacts^12^^,41^. Moreover, ivory working in archaeological sites frequently contain manufacturing debris, such as the cementum-bearing ivory flakes analyzed in this study. This indicates that despite its limited volume per tusk, cementum is accessible in archaeological contexts and represents a realistic and cost-effective target for aDNA analysis. These findings support the development of screening protocols to identify cementum-bearing fragments in archaeological and museum collections.

### Challenges in identifying Magdalenian ivory at Hohle Fels

Although two newly generated mtDNA sequences were initially attributed to Magdalenian layers, direct radiocarbon dating placed both in the Gravettian rather than the Magdalenian. Erosional activity documented at Hohle Fels^42–45^ indicates that the cause for the large absence of archaeological material between the Gravettian and Magdalenian in the record may not be wholly due to settlement preferences. During an intense period of erosion, three major gullies were incised into the Gravettian layers and infilled with Magdalenian material sourcing from the hillside above the cave, falling in through the chimney features towards the back of the cave. Thus, a part of the Magdalenian horizons of Hohle Fels are defined by erosional processes from during and shortly following the Last Glacial Maximum. The ivory samples in this study sourcing from the Magdalenian layers of Hohle Fels map along these erosional gullies (Figure S7), suggesting redeposition of Gravettian ivory into later strata.

Alternatively, the scarcity of Magdalenian ivory may reflect ecological or behavioral factors. Mammoths may have been locally absent from the Ach Valley during this period as suggested by the near absence of mammoth remains in Magdalenian faunal assemblages from the region^46–48^, despite presence in the broader region^49^^,50^. However, isotopic studies of contemporary canid diets indirectly suggest continued consumption of megaherbivores, possibly including mammoths^51^. Nevertheless, Magdalenian groups may have reduced their exploitation of mammoths and their ivory. One possible explanation is tusklessness, a trait known to evolve within as little as 15 years under selective hunting pressure in modern elephants^52^^,53^. Drawings of mammoths such as from the Gönnersdorf Magdalenian do not always depict tusks^54^, while tusks were drawn beyond realistic proportions in earlier cultures^55^ and contemporaneous cultures further afield, such as in Rouffignac cave, France, possibly reflecting tuskless local populations of mammoths. However, even a single tusk or a small tusk could be enough to produce hundreds of ivory beads, and while rare, ivory artifacts directly radiocarbon dated to the Magdalenian have been found at Gönnersdorf^56^. An alternative possibility is that Magdalenian groups in the Ach Valley deliberately chose not to use mammoth ivory in their craftsmanship, favoring other materials such as jet^57–59^. At present, no butchered or securely dated mammoth remains are known from southern Germany that are contemporaneous with the Magdalenian, limiting direct assessment of local mammoth presence. In their absence, the most parsimonious explanations remain either a local disappearance of mammoths from the region around the Ach Valley or a marked reduction in human-mammoth interaction during the Magdalenian.

### Mammoth genetic characterization from anthropogenic remains

By combining genetic sexing, mtDNA lineage analysis, and archaeological context, we provide new insights into the biological traits of mammoths exploited by Upper Paleolithic humans at Hohle Fels. A novel aspect of this study is the successful genetic sex determination of Hohle Fels mammoth remains, revealing a female-biased sex ratio of nearly 3:1 when combining eight newly sequenced and seven previously published samples. This pattern significantly contrasts with naturally accumulated mammoth bonebeds, which are often male-skewed due to natural traps and solitary young males^18^, highlighting a potential human selection effect. Female-leaning ratios are observed in both ivory and bone assemblages independently, indicating that the material type does not substantially bias sex representation, potentially reflecting that the ivory used for artifacts comes from animals procured primarily for subsistence. While we acknowledge that our estimate combines ivory and bone specimens from a large temporal frame, the observed pattern may reflect natural herd structure, as in extant elephants where females dominate social groups^60^, suggesting humans may have preferentially targeted matriarchal herds for predictability or group size. Alternatively, female mammoths may have been selected specifically for symbolic or personal ornamentation artifacts, providing a window into material choice and cultural practices. Further sampling is needed to explore these hypotheses and detect potential period-specific patterns that are not apparent in the current dataset.

By reconstructing twelve new mitogenomes from Hohle Fels ivory, we substantially increase the genetic record of European Late Pleistocene mammoths, nearly doubling the number of known complete Clade III sequences. All newly sequenced specimens fall within mtDNA Clade III, the dominant lineage in Europe prior to the arrival of Clade I between ~ 24.5 and 16.2 ka. While the origin of Clade III remains uncertain due to limited deep-time sampling, the earliest anatomically woolly mammoth, P043Chukochya, carries mtDNA basal to all Clade III lineages but not to Clades I or II, supporting a possible Siberian origin, though gaps in Central Asia and Eastern Europe leave open alternative sources^37,^^61^. The mechanisms behind Clade III’s eventual replacement remain unresolved, with possibilities including climate-driven range shifts, competitive exclusion, or demographic collapse from overhunting. Importantly, these newly generated sequences provide greater temporal and genetic resolution for this lineage, highlighting the value of archaeological ivory in filling gaps where skeletal remains are rare. Continued nuclear genomic analyses and fine-scale dating will be crucial to fully understand the disappearance of Clade III in Europe and its interactions with the emerging Clade I populations.

The mammoth remains from Hohle Fels encompass five distinct sub-clades within Clade III mtDNA diversity. Interestingly, genetically sexed females are represented in three of these sub-clades, indicating that female mammoths were drawn from multiple, genetically distinct maternal lineages. In modern elephants, mtDNA dispersal is extremely limited due to female philopatry, where females typically remain in their natal herds, producing strong matrilineal population structure^62^. If woolly mammoths exhibited similar social behavior, the wide range of mtDNA diversity at Hohle Fels suggests that Upper Paleolithic humans sourced ivory from multiple, genetically distinct mammoth herds. Some of this variation could also reflect temporal changes, since the main archaeological horizons (II, III, IV, and V) each span hundreds of years. However, the phylogeny (Fig. 3) shows that specimens from the same horizon fall into different branches of Clade III, and none of the horizons are restricted to a single sub-clade. This indicates that chronological depth alone cannot explain the observed diversity and instead supports the interpretation that humans repeatedly accessed ivory from different mammoth herds over time, potentially through direct hunting or collection from a wide geographic range. The possibility that mammoth ivory or raw mammoth tusk material was traded or moved between human groups cannot be excluded and may explain the accumulation of such diverse genetic lineages at a single site. One horizon at Hohle Fels (AH Vb) offers a well-preserved context where such questions could be explored in greater detail.

### Genomic potential in the iconic Hohle Fels layer AH Vb

Archaeological Horizon (AH) Vb, the basal Aurignacian layer of Hohle Fels, shows exceptional potential for ancient DNA studies, with ivory piece HOLF022 yielding 20% endogenous DNA despite being from the oldest layer in our dataset. AH Vb is known to be rich in organic preservation and was characterized during excavation as a humid, silty loam with fewer frost fractures than overlying strata^28^. These microenvironmental conditions likely contributed to the exceptional preservation of many iconic finds, such as the female figurine and the flute made from vulture bone, which also originate from this layer^28–30^. The combination of excellent preservation and rich artifact assemblages makes AH Vb an ideal target for future aDNA sampling, enabling detailed investigation of the biological origins of ivory artifacts.

Targeted sampling from AH Vb provides a unique opportunity to determine whether ivory artifacts derive from a single, a few, or many mammoths, and to assess how biological traits such as sex may have influenced human selection. The layer contains complete or refitted ivory figurines and tools, as well as chips and flakes produced during manufacture^12^. These byproducts, alongside finished artifacts, could originate from one or multiple tusks (4,591 kg in total across all Aurignacian horizons, as of 2013)^12^. Biological kinship analyses could link artifacts to an individual or related mammoths in the assemblage, offering critical insights into prehistoric raw material procurement and management strategies. Selection of particular mammoth traits, such as sex, age, herd affiliation, or phenotypic characteristics, may reflect deeper cultural meanings embedded in artifact production and use, particularly in figurines depicting animals or females. Ivory sourced from male or female mammoths could yield different interpretations when used for figurines or symbolic objects. While destructive sampling is unlikely to be feasible for precious artifacts, the development of non-destructive or minimally invasive aDNA methods^63^^,64^ offers new opportunities to study ivory artifacts without compromising cultural heritage.

### Implications for future aDNA studies of ivory

The results presented here demonstrate the broader utility of ancient ivory as a substrate for aDNA research, both at Hohle Fels and across other archaeological contexts. Cementum-targeted sampling consistently yields higher endogenous DNA, and even small fragments or samples with low endogenous content can provide meaningful biological and cultural insights, particularly when combined with targeted mtDNA capture. Collectively, these findings highlight the unique potential of archaeological ivory to inform reconstructions of mammoth biology, herd composition, and population dynamics, as well as human interactions with these animals. By integrating genetic data with archaeological context, ivory artifacts can serve as a powerful lens through which to explore Upper Paleolithic subsistence strategies, material selection, and symbolic behavior, paving the way for future interdisciplinary studies.

## Methods

### Site and sample selection

The site of Hohle Fels (described in more detail in Supplementary Materials) is located in the Ach Valley of the Swabian Jura near Schelklingen, southwestern Germany, a region with a high density of archaeological karstic caves. Because of its rich Paleolithic ivory record and abundance of culturally significant ivory finds, Hohle Fels was selected for this study.

Ivory samples were chosen across the site’s stratigraphy based on size and visible preservation, aiming for two per Upper Paleolithic layer when possible. All pieces are anthropogenic, likely flakes discarded during ivory knapping. Preference was given to pieces containing both cementum and dentin. The final dataset included three Magdalenian, seven Gravettian, and 15 Aurignacian pieces (25 total; Table 1, Figure S1). Four pieces retained intact cementum (HOLF010, HOLF023, HOLF024, HOLF025); the rest were dentin only (Table 1). The city of Schelklingen, as the owner of the site, has granted permission for all analyses of the prehistoric finds, as has the State Office for Cultural Heritage of Baden-Württemberg, which grants permission concerning the scientific excavations. The University of Tübingen is responsible for the investigation of the finds.

No directly dated ivory large enough for internal DNA sampling was available. Instead, radiocarbon dates were obtained for three samples (HOLF001, HOLF003, and HOLF004) with successful mitogenomic construction to at least 90% completeness. The ivory samples were prepared for radiocarbon dating with a collagen extraction from 200 mg of bone powder as described as Protocol F in Cersoy et al.^65^. Such protocols were shown to be sufficient for radiocarbon dating according to a recent study by Talamo et al.^66^. The radiocarbon analysis and calibration were performed at ETH Zurich using OxCal^67^ and the IntCal20 calibration curve^68^.

#### Experimental procedures

Unless specified otherwise, all lab work was performed at the University of Tübingen Institute for Archaeological Sciences in the Archaeo- and Paleogenetic laboratories following aDNA clean lab practices such as wearing body suits, double gloves, a face shield, and cleaning with DNA-away, bleach, and the overnight UV radiation of the entire clean room.

### 3D scanning and sampling

3D scans were created for each piece before destructive sampling. Sampling was conducted in a dedicated clean room using a dental drill. After cleaning the surface with the drill bit, about 50 mg of fine powder was collected from the ivory pieces. Care was taken to minimize heat production by lowering the drill speed and drilling only in short intervals. When possible, only a few holes were drilled in each piece to minimize surface contamination. For very small, thin pieces, a window was created with the drill bit and multiple shallow holes were bored until the target mass was reached. Pieces with both dentin and cementum were sampled for approximately 50 mg of powder each, once for dentin and once for cementum, with full workstation cleaning in between. These paired samples were always processed simultaneously downstream, up to shotgun screening, to minimize batch effects and other external influences.

### DNA extraction, library preparation, mtDNA capture, and sequencing

The collected ivory powder was extracted for DNA following the silica-based procedure from Dabney et al.^69^, which is optimized for the recovery of short DNA fragments, along with positive and negative extraction controls with minor adjustments^70^. The extracts underwent partial USER treatment^71^ and converted into double stranded libraries with dual indexes following Meyer and Kircher^72^ and Kircher et al.^73^ along with positive and negative library controls. Libraries were quantified with qPCR twice, once after library preparation to quantify library copy number before any amplification and once after indexing PCRs in preparation for sequencing.

To prepare for mtDNA capture, a portion of the indexed libraries were initially amplified to reach a concentration > 200 ng/uL and then pooled into a maximum of five samples (of similar copy numbers as calculated by qPCR directly following library preparation) per pool to a maximum of 2 µg concentration total per pool in a volume of 10 µL, with water making up the remaining volume. Blanks were pooled together in their own pool. Positive controls from library and extract were not included, instead, at this point, two unenriched previously amplified indexed mammoth libraries (TU175, TU176) were pooled together for capture to act as capture positive controls. This resulted in nine pools that were then subjected to bead-based in-solution mtDNA capture with baits generated in Fellows Yates et al.^17^ and immortalized based on the protocol described in the SI of Furtwängler et al.^74^. The products were purified with the MinElute kit following manufacturer protocol.

Samples were diluted to 10 nM according to TapeStation quantification and pooled according to lane space before being sent to the Max Planck Institute for Evolutionary Anthropology in Leipzig for sequencing. The pools were sent for single-end sequencing both shotgun and mtDNA capture in two separate lanes on an Illumina HiSeq4000 platform for 1 × 75 cycles at a depth of ~ 3 million reads per sample. Blanks were also sent for sequencing at 1 × 75 on an Illumina NextSeq platform. Selected mtDNA captured samples and unenriched libraries were additionally sent for deeper sequencing on the same Illumina HiSeq4000 machine for 1 × 75 cycles. Parameters for deeper sequencing of selected capture pools were determined based on initial sequencing results for optimal sample enhancement and recovery between 3 and 12 million reads.

### Read processing, consensus calling, and alignment

The merged raw reads were processed separately for shotgun and capture data using the automated pipeline manager EAGER version 1.92.56 (Efficient Ancient Genome Reconstruction)^75^. Sequences of captured libraries were mapped only against the woolly mammoth mitochondrial genome (GenBank: NC007596.2)^76^ while unenriched shotgun sequencing data was mapped against both the woolly mammoth mitochondrial genome and the African savannah elephant nuclear genome LoxAfr4 (The Broad Institute)^77^.

Mitochondrial DNA consensus sequences were called on the processed and trimmed bam files (2-bp at both molecule termini) using the Geneious (https://www.geneious.com) consensus extraction function. Bases were called at sites with at least three non-duplicated reads where the majority base was present at a frequency higher than 50%, all other bases were called as Ns. Samples with less than 90% of sites covered in the consensus were excluded from the multiple genome alignments.

Other recently published proboscidean mitochondrial genomes with less than 10% missing sites were collected from GenBank (see Supplementary Table S9 in the Appendix for full list and GenBank accessions) using efetch^78^ and alignment to the newly generated mitogenomes. Multiple genome alignments were carried out in MUSCLE v.3.8.1551^35^ with 2 maximum iterations. An Asian elephant mitogenome was included in the alignment to serve as an outgroup for tree rooting (Asian Elephant, accession: EF588275).

### Phylogenetic data analysis

Trees were constructed from the aligned mitogenomes to visualize evolutionary patterns and mtDNA shifts. Initially, the names of the fasta files were changed to more user-friendly identifiers using the rename.fasta() function in phylotools^79^ in R v.4.0.2^80^. Phylogenetic trees were constructed in MEGA 11.0.8^36^ using the Maximum Parsimony method with partial deletion of 80% and 1000 bootstrap iterations. The resulting MEGA trees were converted to Newick format and imported into FigTree v1.4.4 (http://tree.bio.ed.ac.uk/software/figtree/) for visualization adjustments.

### Sexing

Sexes were determined from the trimmed bam files of the shotgun sequence reads after mapping to the nuclear genome LoxAfr4. As this reference genome is sourced from a female elephant, there is no reference to a Y chromosome (ChrY). Instead of using ChrY as a proxy, chromosome 8 (Chr8), a chromosome of similar size to chromosome X (ChrX), was used for comparison following Pečnerová et al.^18^. Because male mammoths only have one X chromosome, they are expected to have a ChrX: Chr8 ratio of approximately 0.5, while females are expected to have a ChrX: Chr8 ratio of approximately 1. The reads mapping to ChrX and Chr8 were counted using the xykaryotyper.py script from Skoglund et al.^81^. Standard errors were estimated according to^18^. A two-tailed binomial test was used to test if the resulting sex ratio varied significantly from the null hypothesis of 1:1 in R. Fisher’s exact test was used to compare ratios to one another.

### Material comparison

The metrics for “mammoth DNA,” “informative sequences per mg,” “genomic coverage,” and “cost effectiveness” were used to assess different aspects of aDNA acquisition success from Hohle Fels ivory, following Parker et al.^34^. The metric “length” was simply the average mapped fragment lengths for each sample. The total DNA molecules in a library was calculated with qPCR on libraries prior to indexing for a 40 uL library. The other metrics and their dependencies were calculated as follows:

Proportion Mammoth DNA Recovered from each sampling effort was calculated as:

$$\:\frac{Total\:number\:of\:mapped\:reads\:before\:duplicate\:removal\:and\:before\:quality\:filtering}{Total\:number\:of\:reads\:after\:merging\:and\:filtering\:for\:quality\:and\:length}\:$$

Post Capture Enrichment Factor for on-target mtDNA reads was calculated as:

$$\:\frac{Proportion\:Mammoth\:DNA\:with\:Enrichment}{Proportion\:Mammoth\:DNA\:without\:Enrichment}$$

The number of Informative sequences in a library per mg sample input

$$\:\frac{No.\:of\:DNA\:Molecules\:in\:Library\:*\:Number\:of\:mapped\:reads\:after\:duplicate\:removal\:and\:quality\:filtering}{Total\:number\:of\:reads\:after\:merging\:and\:filtering\:for\:quality\:and\:length\:*\:mg\:Input}$$

The total genomic coverage gained per 50 mg sample input was estimated as:

$$\:\frac{No.\:of\:DNA\:Molecules\:in\:Library\:*\:Prop.\:Mammoth\:DNA\:\:*\:Avg.\:Mapped\:Read\:Length}{\:Length\:of\:Target\:Genome}\:\mathrm{*}\:\frac{uL\:Extract\:total\:*\:50\:mg}{\:uL\:Extract\:used\:*\:mg\:Input}$$

The cost-effectiveness, or the number of unique base pairs gained from one million reads of sequencing effort per milligram of sample input, was estimated as:

$$\:\frac{{Unique\:Mapped\:\operatorname{Re} ads\:*\:Avg\:Mapped\:\operatorname{Re} ad\:Length\:\:}}{{Total\:Raw\:\operatorname{Re} ads\:Sequencing\:Effort\:*\:mg\:Input}}*1,000,000\;$$

raw reads sequencing effort

Mean coverage (fold) was calculated as:

$$\:\frac{Total\:Mapped\:Reads\:*Avg\:Mapped\:Read\:Length}{Length\:of\:Target\:Genome}$$

For the four individuals with both dentin and cementum components (HOLF010, HOLF023, HOLF024, and HOLF025), these values were calculated for both materials and treated as paired by individual. To test if there was a significant difference between the calculation results of the two conditions, a paired t-test was run in R. Paired t-tests can only be run meaningfully if the differences are normally distributed. Due to the low sample size, the Shapiro-Wilk normality test was utilized.

## Supplementary Information

Below is the link to the electronic supplementary material.

Supplementary Material 1

Supplementary Material 2

## Data Availability

The nuclear and mitochondrial DNA mapped sequences of the analyzed individuals in this study are available at the European Nucleotide Archive (ENA) under study accession number PRJEB103048. The twelve nearly complete mtDNA sequences reconstructed in this work are deposited in GenBank under the accession numbers PZ244049 to PZ244060.

## References

[1] Terrible Beauty,. *Elephant - Human - Ivory* (Hirmer, 2021).

[2] Beuckers, K. G. *Mittelalterliche Elfenbeinarbeiten aus der Sammlung des Badischen Landesmuseums Karlsruhe* (Badisches Landesmuseum, 1999).

[3] Gvozdover, M. *Art of the Mammoth Hunter* (Oxbow Books, 1995).

[4] Khlopachev, G. A. Upper Paleolithic images, symbols, signs. Catalog of small-form art objects and unique finds of the Upper Paleolithic from archaeological collections of MAE RAS. (2016).

[5] Pitulko, V. V. et al. Paleoanthropology. Early human presence in the Arctic: Evidence from 45,000-year-old mammoth remains. *Science* **351**, 260–263 (2016). 26816376 10.1126/science.aad0554

[6] Wolf, S. & Heckel, C. Ivory ornaments of the Aurignacian in Western Europe: Case studies from France and Germany. *Anthropologie* **122**, 348–373 (2018).

[7] Heckel, C. E. Reconsidering production organization in the Early Upper Palaeolithic: The case for specialized production of Aurignacian beads. *Quat. Int.* **491**, 11–20 (2018).

[8] Heckel, C. E. & Wolf, S. Ivory debitage by fracture in the Aurignacian: Experimental and archaeological examples. *J. Archaeol. Sci.* **42**, 1–14 (2014).

[9] Antl, W. & Bosch, M. D. The use of ivory at the Gravettian site Grub/Kranawetberg, Lower Austria. *Anthropologie (1962-)* **53**, 233–234 (2015).

[10] Heckel, C. Creating wealth in the ice age: Ivory beads of the French Aurignacian. *Paléo*10.4000/paleo.6952 (2021).

[11] Heckel, C., Wolf, S., THE CIRCULATION OF & ORNAMENTS IN AURIGNACIAN CONTEXTS. in *Contact, Circulation, Exchange: Proceedings of the Modified Bone & Shell UISPP Commission Conference (2–3 March* University of Trnava) 13 (Archaeopress Publishing Ltd, 2023). (2017).

[12] Wolf, S. *Schmuckstücke : die Elfenbeinbearbeitung im Schwäbischen Aurignacien* (Kerns, 2015).

[13] Schwarz, C. et al. New insights from old bones: DNA preservation and degradation in permafrost preserved mammoth remains. *Nucleic Acids Res.* **37**, 3215–3229 (2009). 19321502 10.1093/nar/gkp159 PMC2691819

[14] Dalén, L., Heintzman, P. D., Kapp, J. D. & Shapiro, B. Deep-time paleogenomics and the limits of DNA survival. *Science* **382**, 48–53 (2023). 37797036 10.1126/science.adh7943 PMC10586222

[15] Palkopoulou, E. et al. Holarctic genetic structure and range dynamics in the woolly mammoth. *Proceedings of the Royal Society B: Biological Sciences* **280**, 20131910 (2013). 10.1098/rspb.2013.1910 PMC3779339 24026825

[16] Chang, D. et al. The evolutionary and phylogeographic history of woolly mammoths: A comprehensive mitogenomic analysis. *Sci. Rep.* **7**, 44585 (2017). 28327635 10.1038/srep44585 PMC5361112

[17] Fellows Yates, J. A. et al. Central European Woolly Mammoth Population Dynamics: Insights from Late Pleistocene Mitochondrial Genomes. *Sci. Rep.* **7**, 17714 (2017). 29255197 10.1038/s41598-017-17723-1 PMC5735091

[18] Pečnerová, P. et al. Genome-based sexing provides clues about behavior and social structure in the Woolly Mammoth. *Curr. Biol.* **27**, 3505-3510e.e3 (2017). 29103934 10.1016/j.cub.2017.09.064

[19] Rey-Iglesia, A. et al. Ancient biomolecular analysis of 39 mammoth individuals from Kostenki 11-Ia elucidates Upper Palaeolithic human resource use. *Quaternary Environments and Humans* **3**, 100049 (2025).

[20] Drucker, D. G. et al. Tracking possible decline of woolly mammoth during the Gravettian in Dordogne (France) and the Ach Valley (Germany) using multi-isotope tracking (13C, 14C, 15N, 34S, 18O). *Quat. Int.* **359–360**, 304–317 (2015).

[21] White, R. Systems of Personal Ornamentation in the Early Upper Palaeolithic: Methodological Challenges and New Observations. *Rethinking Hum. Revolution: New. Beahavioural Biol. Perspect. Origin Dispersal Mod. Humans* 287–302 (2007).

[22] Winters, M. et al. Isolation of DNA from small amounts of elephant ivory: Sampling the cementum with total demineralization extraction. *Forensic Sci. Int.* **288**, 131–139 (2018). 29753151 10.1016/j.forsciint.2018.04.036

[23] Mailand, C. & Wasser, S. K. Isolation of DNA from small amounts of elephant ivory. *Nat. Protoc.* **2**, 2228–2232 (2007). 17853880 10.1038/nprot.2007.318

[24] Adler, C. J., Haak, W., Donlon, D. & Cooper, A. Survival and recovery of DNA from ancient teeth and bones. *J. Archaeol. Sci.* **38**, 956–964 (2011).

[25] Damgaard, P. B. et al. Improving access to endogenous DNA in ancient bones and teeth. *Sci. Rep.* **5**, 11184 (2015). 26081994 10.1038/srep11184 PMC4472031

[26] Higgins, D., Kaidonis, J., Townsend, G., Hughes, T. & Austin, J. J. Targeted sampling of cementum for recovery of nuclear DNA from human teeth and the impact of common decontamination measures. *Investig. Genet.* **4**, 18 (2013). 24139166 10.1186/2041-2223-4-18 PMC3853689

[27] Higgins, D., Rohrlach, A. B., Kaidonis, J., Townsend, G. & Austin, J. J. Differential nuclear and mitochondrial DNA preservation in post-mortem teeth with implications for forensic and ancient DNA studies. *PLoS One* **10**, e0126935 (2015). 25992635 10.1371/journal.pone.0126935 PMC4438076

[28] Conard, N. J., Malina, M. & Münzel, S. C. New flutes document the earliest musical tradition in southwestern Germany. *Nature* **460**, 737–740 (2009). 19553935 10.1038/nature08169

[29] Conard, N. J. A female figurine from the basal Aurignacian of Hohle Fels Cave in southwestern Germany. *Nature* **459**, 248–252 (2009). 19444215 10.1038/nature07995

[30] Conard, N. J. Palaeolithic ivory sculptures from southwestern Germany and the origins of figurative art. *Nature* **426**, 830–832 (2003). 14685236 10.1038/nature02186

[31] Dutkiewicz, E., Wolf, S., Floss, H. & Conard, N. J. Les objets en ivoire du Jura souabe. *Anthropologie* **122**, 447–468 (2018).

[32] Conard, N. J., Kitagawa, K., Krönneck, P., Böhme, M. & Münzel, S. C. The Importance of Fish, Fowl and Small Mammals in the Paleolithic Diet of the Swabian Jura, Southwestern Germany. In *Zooarchaeology and Modern Human Origins* (eds Clark, J. L. & Speth, J. D.) 173–190 (Springer, 2013).

[33] Münzel, S. C., Wolf, S., Drucker, D. G. & Conard, N. J. The exploitation of mammoth in the Swabian Jura (SW-Germany) during the Aurignacian and Gravettian period. *Quat. Int.* **445**, 184–199 (2017).

[34] Parker, C. et al. A systematic investigation of human DNA preservation in medieval skeletons. *Sci. Rep.* **10**, 1–16 (2020). 33106554 10.1038/s41598-020-75163-w PMC7588426

[35] Edgar, R. C. MUSCLE: Multiple sequence alignment with high accuracy and high throughput. *Nucleic Acids Res* **32**, 1792–1797 (2004). 15034147 10.1093/nar/gkh340 PMC390337

[36] Tamura, K., Stecher, G. & Kumar, S. MEGA11: Molecular Evolutionary Genetics Analysis version 11. *Mol. Biol. Evol.*10.1093/molbev/msab120 (2021). 33892491 10.1093/molbev/msab120 PMC8233496

[37] Chacón-Duque, J. C. et al. A million years of mammoth mitogenome evolution. *Mol. Biol. Evol.* **42**, msaf065 (2025). 40202893 10.1093/molbev/msaf065 PMC11980863

[38] Cui, F. Z., Wen, H. B., Zhang, H. B., Ma, C. L. & Li, H. D. Nanophase hydroxyapatite-like crystallites in natural ivory. *J. Mater. Sci. Lett.* **13**, 1042–1044 (1994).

[39] Locke, M. Structure of ivory. *J. Morphol.* **269**, 423–450 (2008). 18157860 10.1002/jmor.10585

[40] Baker, B. W., Jacobs, R. L., Mann, M. J., Espinoza, E. O. & Grein, G. *Identification Guide for Ivory and Ivory Substitutes* (World Wildlife Fund Inc., 2020).

[41] Riek, G. Die Eiszeitjägerstation am Vogelherd in Lonetal. *Die Kulturen Tübingen*. **1**, 338 (1934).

[42] Barbieri, A. et al. Interpreting gaps: A geoarchaeological point of view on the Gravettian record of Ach and Lone valleys (Swabian Jura, SW Germany). *J. Archaeol. Sci.* **127**, 105335 (2021).

[43] Barbieri, A. *Landscape Changes, Cave Site Formation and Human Occupation during the Late Pleistocene: A Geoarchaeological Study from the Ach and Lone Valleys (Swabian Jura, SW Germany)*. (2017).

[44] Miller, C. E. *A Tale of Two Swabian Caves - Geoarchaeological Investigations at Hohle Fels and Geißenklösterle* (Kerns Verlag, 2015).

[45] Taller, A. *Das Magdalénien des Hohle Fels* (Kerns Verlag, 2014).

[46] Boessneck, J. & den Driesch, A. Die junglpleistozänen Tierknochenfunde aus der Brillenhöhle.-Forsch. *Berichte Vor-u. Frühgesch. Baden-Württemberg* **4**, 34–49 (1973).

[47] Münzel, S. C. Die jungpleistozäne Großsäugerfauna aus dem Geißenklösterle. In *Geißenklösterle. Chronostratigraphie, Paläoumwelt und Subsistenz im Mittel- und Jungpaläolithikum der Schwäbischen Alb* (eds Conard, N. J. et al.) 147–415 (Kerns Verlag, 2019).

[48] Napierala, H., Münzel, S. C. & Conard, N. J. Die Fauna des Magdalénien vom Hohle Fels In (ed. Taller, A.) (2014).

[49] Stuart, A. J., Sulerzhitsky, L. D., Orlova, L. A., Kuzmin, Y. V. & Lister, A. M. The latest woolly mammoths (*Mammuthus primigenius* Blumenbach) in Europe and Asia: A review of the current evidence. *Quat. Sci. Rev.* **21**, 1559–1569 (2002).

[50] Napierala, H. Die Tierknochen aus dem Kesslerloch. Neubearbeitung der paläolithischen Fauna. *Beiträge zur Schaffhauser Archäologie 2.* stamm + co AG, (2008).

[51] Baumann, C. et al. OPEN A refined proposal for the origin of dogs: the case study of Gnirshöhle, a Magdalenian cave site. *Sci. Rep.* **11**, 1–14 (2021). 33664287 10.1038/s41598-021-83719-7 PMC7933181

[52] Chiyo, P. I., Obanda, V. & Korir, D. K. Illegal tusk harvest and the decline of tusk size in the African elephant. *Ecol. Evol.* **5**, 5216–5229 (2015). 30151125 10.1002/ece3.1769 PMC6102531

[53] Campbell-Staton, S. C. et al. Ivory poaching and the rapid evolution of tusklessness in African elephants. *Science* **374**, 483–487 (2021). 34672738 10.1126/science.abe7389

[54] Bosinski, G. Die Ausgrabungen in Gönnersdorf 1968–1976 und die Siedlungsbefunde der Grabung 1968. *Der Magdalénian-Fundplatz Gönnersdorf*10.11588/bjb.1984.0.67269 (1979).

[55] Le Guillou, Y. The sacristy of the Chauvet Cave and its access corridor. *Bulletin de la Société Préhistorique Française Ariège-Pyrénées* LVI, (2001).

[56] Stevens, R. E. et al. Major excursions in sulfur isotopes linked to permafrost change in Eurasia during the last 50,000 years. *Nature Geoscience* **18**, 961–965 (2025). 41079034 10.1038/s41561-025-01760-x PMC12510881

[57] Eriksen, B. V. *Change and Continuity in a Prehistoric Hunter-Gatherer Society: a study of cultural adaptation in late glacial - early postglacial southwestern Germany* (Verlag Archaeologica Venatoria, 1991).

[58] Peters, E. & Toepfer, V. Der Abschluß der Grabungen am Petersfels bei Engen im badischen Hegau. *Prähist. Z.* **23**, 155–199 (1932).

[59] Wolf, S. Bemalte Steine aus dem Magdalénien der Hohle Fels Höhle bei Schelklingen. *Laichinger Höhlenfreund* 51–62 (2019).

[60] Goswami, V. R. et al. Towards a reliable assessment of Asian elephant population parameters: The application of photographic spatial capture–recapture sampling in a priority floodplain ecosystem. *Sci. Rep.* **9**, 8578 (2019). 31189980 10.1038/s41598-019-44795-y PMC6561924

[61] van der Valk, T. et al. Million-year-old DNA sheds light on the genomic history of mammoths. *Nature* **591**, 265–269 (2021). 33597750 10.1038/s41586-021-03224-9 PMC7116897

[62] Ishida, Y., Georgiadis, N. J., Hondo, T. & Roca, A. L. Triangulating the provenance of African elephants using mitochondrial DNA. *Evolutionary Applications* **6**, 253–265 (2013). 23798975 10.1111/j.1752-4571.2012.00286.x PMC3689351

[63] Harney, É. et al. A minimally destructive protocol for DNA extraction from ancient teeth. *Genome Res.* **31**, 472–483 (2021). 33579752 10.1101/gr.267534.120 PMC7919446

[64] Essel, E. et al. Ancient human DNA recovered from a Palaeolithic pendant. *Nature* **618**, 328–332 (2023). 37138083 10.1038/s41586-023-06035-2 PMC10247382

[65] Cersoy, S., Zazzo, A., Lebon, M., Rofes, J. & Zirah, S. Collagen Extraction and Stable Isotope Analysis of Small Vertebrate Bones: A Comparative Approach. *Radiocarbon* **59**, 679–694 (2017).

[66] Talamo, S., Fewlass, H., Maria, R. & Jaouen, K. Here we go again’: The inspection of collagen extraction protocols for ^14^ C dating and palaeodietary analysis. *STAR Sci. Technol. Archaeol. Res.* **7**, 62–77 (2021). 34381618 10.1080/20548923.2021.1944479 PMC8300532

[67] Ramsey, C. B. Bayesian analysis of radiocarbon dates. *Radiocarbon* **51**, 337–360 (2009).

[68] Reimer, P. J. et al. The IntCal20 Northern Hemisphere Radiocarbon Age Calibration Curve (0–55 cal kBP). *Radiocarbon* **62**, 725–757 (2020).

[69] Dabney, J. et al. Complete mitochondrial genome sequence of a Middle Pleistocene cave bear reconstructed from ultrashort DNA fragments. *Proc. Natl. Acad. Sci. U. S. A.* **110**, 15758–15763 (2013). 24019490 10.1073/pnas.1314445110 PMC3785785

[70] Korlević, P. et al. Reducing microbial and human contamination in DNA extractions from ancient bones and teeth. *Biotechniques* **59**, 87–93 (2015). 26260087 10.2144/000114320

[71] Rohland, N., Harney, E., Mallick, S., Nordenfelt, S. & Reich, D. Partial uracil-DNA-glycosylase treatment for screening of ancient DNA. *Philos. Trans. R. Soc. Lond. B Biol. Sci.*10.1098/rstb.2013.0624 (2015). 25487342 10.1098/rstb.2013.0624 PMC4275898

[72] Meyer, M. & Kircher, M. Illumina Sequencing Library Preparation for Highly Multiplexed Target Capture and Sequencing. *Cold Spring Harb. Protoc.* **2010**(6), db.prot5448 (2010). 10.1101/pdb.prot5448 20516186

[73] Kircher, M., Sawyer, S. & Meyer, M. Double indexing overcomes inaccuracies in multiplex sequencing on the Illumina platform. *Nucleic Acids Res.* **40**, e3–e3 (2012). 22021376 10.1093/nar/gkr771 PMC3245947

[74] Furtwängler, A. et al. Ratio of mitochondrial to nuclear DNA affects contamination estimates in ancient DNA analysis. *Sci. Rep.* **8**, 14075 (2018). 30232341 10.1038/s41598-018-32083-0 PMC6145933

[75] Peltzer, A. et al. EAGER: Efficient ancient genome reconstruction. *Genome Biol* **17**, 60 (2016). 27036623 10.1186/s13059-016-0918-z PMC4815194

[76] Krause, J. et al. Multiplex amplification of the mammoth mitochondrial genome and the evolution of Elephantidae. *Nature* **439**, 724–727 (2006). 16362058 10.1038/nature04432

[77] Palkopoulou, E. et al. A comprehensive genomic history of extinct and living elephants. *Proc. Natl. Acad. Sci. U. S. A.* **115**, E2566–E2574 (2018). 29483247 10.1073/pnas.1720554115 PMC5856550

[78] Kans, J. *Entrez Direct: E-Utilities on the Unix Command Line,* National Center for Biotechnology Information (US), (2022).

[79] Zhang, J. *Phylotools: Phylogenetic Tools for Eco-Phylogenetics*. (2017).

[80] R Core Team. *R: A Language and Environment for Statistical Computing* (R Foundation for Statistical Computing, 2018).

[81] Skoglund, P., Storå, J., Götherström, A. & Jakobsson, M. Accurate sex identification of ancient human remains using DNA shotgun sequencing. *J. Archaeol. Sci.* **40**, 4477–4482 (2013).

