# Supplementary material for: Ancient DNA from the Upper Paleolithic mammoth ivory of Hohle Fels, Germany
Source: Sci Rep. 2026 May 14;16:15181. doi: 10.1038/s41598-026-46761-x (PMC13176336; doi:10.1038/s41598-026-46761-x)
Supplement: Supplementary file 1 — Supplementary Material 1 [file 41598_2026_46761_MOESM1_ESM.docx]

**Supplementary Text**

**Supplementary Note 1: Hohle Fels Archaeological Contexts**

The site of Hohle Fels is located in the Ach Valley in Southwestern Germany’s Swabian Jura, which hosts a high density of archaeological karstic cave sites. The Ach Valley is the historic path of the Danube River, and the Ach River feeds into the modern path of the Danube in Ulm, about 15 km east of Hohle Fels. Standing 534 m above sea level, Hohle Fels, the largest cave site in the Ach Valley (c. 6000 m3) and among the largest in the region, is known for its spectacular very early Aurignacian finds, including mammoth ivory carvings, such as the earliest female figurine[^1^](https://paperpile.com/c/k5bpNx/YAPR), and the earliest evidence of musical instruments[^2^](https://paperpile.com/c/k5bpNx/a8CH). Excavations of the cave by Fraas and Hartmann began in 1870, the earliest Paleolithic research documented in the Ach Valley. Most of the finds from this period were lost in World War II. Research at the site subsequently continued with excavations run by Riek, Hahn, and then Conard, which are now ongoing[^3^](https://paperpile.com/c/k5bpNx/AXr8); excavation reports published annually in Ausgrabungen in Baden-Württemberg[^4–21^](https://paperpile.com/c/k5bpNx/0v2F+ggzL+2okU+pksA+ZD3K+y6Xd+iHdx+NTiX+J7gA+q9XK+q2RQ+yZdW+qnum+S5vU+Vk0C+MW9I+8hIB+I9Aq).

Hohle Fels’s excavated archaeological sequence spans from the Middle Paleolithic to the Magdalenian, with Middle Paleolithic (AH VI+) dating to older than ~42 ky cal BP, Aurignacian layers (AH Vb – IIe) dating to 42 – 35 ky cal BP, Gravettian layers (AH IIc – IIb) dating to 35 – 28 ky cal BP[^1,22–25^](https://paperpile.com/c/k5bpNx/55Fo+YAPR+w7Uo+Si3f+5nbU), with AH IId identified as a transition layer between the Gravettian and Aurignacian, and Magdalenian layers (AH IIab – I) dating to 17 – 13 ky cal BP. Caves in the Ach and Lone Valleys show an archaeological record suggesting both intense periods of human occupation interspersed by nearly sterile deposits, but the two valleys do not have temporally identical occupation records and gaps despite their close proximity. These gaps, and how they differed by site and valley, had been previously interpreted as differential settlement strategies [^23,24,26,27^](https://paperpile.com/c/k5bpNx/Si3f+w7Uo+MOTS+tkGd), but recent geoarchaeological analyses have provided strong evidence to suggest that the gaps may be the result of significant erosion during and following the LGM. Erosional processes could have impacted each site differently due to variation in environmental conditions, such as altitude and/or slope[^28^](https://paperpile.com/c/k5bpNx/fPcK). In regards to Hohle Fels specifically, both sediments and archaeological material from about 28 – 17 ky cal BP are largely absent from the cave’s stratigraphy. During an intense period of erosion, three major gullies were incised into the Gravettian layers and infilled with Magdalenian material sourcing from the hillside above the cave, falling in through the chimney features towards the back of the cave. Thus, the Magdalenian horizons of Hohle Fels are defined by erosional processes from during and shortly following the LGM. The ivory samples in this study sourcing from the Magdalenian layers of Hohle Fels map along these erosional gullies (Figure S7).

In 2014, Riehl et al.[^29^](https://paperpile.com/c/k5bpNx/DfAE) reconstructed a cold local climate dominated by pine trees during the Gravettian and Aurignacian occupations of Hohle Fels with slight warming during the end of the Aurignacian-Gravettian transition using charcoal, pollen, and phytolith analysis. In the Magdalenian, the area was likely similar to a modern tundra ecosystem with warmer winters, more annual precipitation, and longer vegetative activity periods, resulting in a mosaic with patches of trees[^30^](https://paperpile.com/c/k5bpNx/N4D3).

**Supplementary Note 2: The exceptional DNA preservation of HOLF022**

Based on endogenous DNA after shotgun sequencing, one sample from the lowest Aurignacian horizon of Hohle Fels (AH Vb), HOLF022, had the best biomolecular preservation (Table 5). Layer Vb of Hohle Fels has been known for its exceptional ivory finds. Both the female figurine and flute fragments of Hohle Fels were found in Layer Vb, representing the current oldest female figurine that has ever been found and the oldest evidence of human musical instruments[^1,2^](https://paperpile.com/c/k5bpNx/YAPR+a8CH). This muddy layer has been dated to start around 41.7 ky cal BP[^22^](https://paperpile.com/c/k5bpNx/55Fo) and is thought to be characterized by a warm phase that was shortly followed by a colder period. It is unexpected that a sample from the lowest layer would have the best endogenous DNA yield (12.94x more nucDNA percentage in shotgun sequencing than the next highest sample). Such a well-preserved specimen without enrichment shows high potential for detailed DNA work in the future, such as the sequencing of the entire genome. Two samples (HOLF021 and HOLF022) were both sequenced from AH Vb and successfully produced high quality mitogenomes, suggesting the viability of aDNA studies from ivory in Hohle Fels AH Vb.

**References**

1. [Conard, N. J. A female figurine from the basal Aurignacian of Hohle Fels Cave in southwestern Germany. *Nature* **459**, 248–252 (2009).](http://paperpile.com/b/k5bpNx/YAPR)

2. [Conard, N. J., Malina, M. & Münzel, S. C. New flutes document the earliest musical tradition in southwestern Germany. *Nature* **460**, 737–740 (2009).](http://paperpile.com/b/k5bpNx/a8CH)

3. [Münzel, S. C. & Conard, N. J. Cave bear hunting in Hohle Fels Cave in the Ach Valley of the Swabian Jura. (2004) doi:](http://paperpile.com/b/k5bpNx/AXr8)[10.13140/RG.2.1.2271.0249](http://dx.doi.org/10.13140/RG.2.1.2271.0249)[.](http://paperpile.com/b/k5bpNx/AXr8)

4. [Conard, N., Langguth, K. & Uerpmann, H.-P. Einmalige Funde aus dem Aurignacien und erste Belege für ein Mittelpaläolithikum im Hohle Fels bei Schelklingen, Alb-Donau-Kreis. *Archäologische Ausgrabungen in Baden-Württemberg* 21–27 (2002).](http://paperpile.com/b/k5bpNx/0v2F)

5. [Conard, N. J., Langguth, K. & Uerpmann, H.-P. Die Grabungen 1999 in den Gravettien-Schichten des Hohle Fels bei Schelklingen, Alb-Donau-Kreis. *Archäologische Ausgrabungen in Baden-Württemberg* 21–25 (1999).](http://paperpile.com/b/k5bpNx/ggzL)

6. [Conard, N. J., Langguth, K. & Uerpmann, H.-P. Die Ausgrabungen im Gravettien des Hohle Fels bei Schelklingen, Alb-Donau-Kreis. *Archäologische Ausgrabungen in Baden-Württemberg* 18–22 (2000).](http://paperpile.com/b/k5bpNx/2okU)

7. [Conard, N. J., Langguth, K. & Uerpmann, H.-P. Neue Aurignacien-Fundschichten im Hohle Fels bei Schelklingen, Alb-Donau-Kreis. *Archäologische Ausgrabungen in Baden-Württemberg* 21–26 (2001).](http://paperpile.com/b/k5bpNx/pksA)

8. [Conard, N. J., Langguth, K. & Uerpmann, H.-P. Die Ausgrabungen 2003 in den Gravettien-und Aurignacien Schichten des Hohle Fels bei Schelklingen, Alb-Donau-Kreis, und die kulturelle Entwicklung im frühen Jungpaläolithikum. *Archäologische Ausgrabungen in Baden-Württemberg* 17–22 (2003).](http://paperpile.com/b/k5bpNx/ZD3K)

9. [Conard, N. J. & Janas, A. Ausgrabungen im Hohle Fels: Fundschichten aus dem Mittelpaläolithikum und Neues zur Jagdtechnik der Neandertaler. in *Archäologische Ausgrabungen Bad Württemb 2020* 60–65 (2021).](http://paperpile.com/b/k5bpNx/y6Xd)

10. [Conard, N. J. & Malina, M. Die Ausgrabungen 2004 in den frühen jungpaläolithischen Schichten des Hohle Fels bei Schelklingen, Alb-Donau-Kreis. pro ano. *Archäologische Ausgrabungen in Baden-Württemberg* 17–21 (2004).](http://paperpile.com/b/k5bpNx/iHdx)

11. [Conard, N. J. & Malina, M. Neue Ergebnisse zum Mittelpaläolithikum, zum Aurignacien und zu den letzen Neandertalern am Hohle Fels bei Schelklingen, Alb-Donau-Kreis. *Archäologische Ausgrabungen in Baden-Württemberg* 17–20 (2005).](http://paperpile.com/b/k5bpNx/NTiX)

12. [Conard, N. J. & Malina, M. Die Ausgrabung 2007 im Hohle Fels bei Schelklingen, Alb-Donau-Kreis, und neue Einblicke in die Anfänge des Jungpaläoloithikums. *Archäologische Ausgrabungen in Baden-Württemberg* **2007**, 17–20 (2007).](http://paperpile.com/b/k5bpNx/J7gA)

13. [Conard, N. J. & Malina, M. Spektakuläre Funde aus dem unteren Aurignacien vom Hohle Fels bei Schelklingen, Alb-Donau-Kreis. *Archäologische Ausgrabungen in Baden-Württemberg* 19–22 (2008).](http://paperpile.com/b/k5bpNx/q9XK)

14. [Conard, N. J. & Malina, M. Neue Forschungen in den Magdalénien-schichten des Hohle fels bei Schelklingen. *Archäologische Ausgrabungen in Baden-Württemberg* **2011**, 56–60 (2011).](http://paperpile.com/b/k5bpNx/q2RQ)

15. [Conard, N. J. & Malina, M. Grabungen in Schichten des Moustérien und Gravettien im Hohle Fels bei Schelklingen. *Archäologische Ausgrabungen in Baden-Württemberg* 78–83 (2012).](http://paperpile.com/b/k5bpNx/yZdW)

16. [Conard, N. J. & Malina, M. Vielfältige Funde aus dem Aurigancien und ein bemalter Stein aus dem Magdalénien vom Hohle Fels bei Schelklingen. *Archäologische Ausgrabungen in Baden-Württemberg* 58–63 (2013).](http://paperpile.com/b/k5bpNx/qnum)

17. [Conard, N. J. & Malina, M. Eine mögliche zweite Frauenfigurine vom Hohle Fels und Neues zur Höhlennutzung im Mittel-und Jungpaläolithikum: Schelklingen, Alb-Donau-Kreis. *Archäologische Ausgrabungen in Baden-Württemberg* 54–59 (2014).](http://paperpile.com/b/k5bpNx/S5vU)

18. [Conard, N. J. & Malina, M. Außergewöhnliche neue Funde aus den aurignacienzeitlichen Schichten vom Hohle Fels bei Schelklingen. *Archäologische Ausgrabungen in Baden-Württemberg* **2015**, 60–66 (2015).](http://paperpile.com/b/k5bpNx/Vk0C)

19. [Conard, N. J. & Malina, M. Weiterführende Ausgrabungen im Hohle Fels und neue Einblicke in die Nutzung von Ocker im Jungpaläolithikum. *Archäologische Ausgrabungen in Baden-Württemberg* 56–59 (2018).](http://paperpile.com/b/k5bpNx/MW9I)

20. [Conard, N. J. & Malina, M. Fortsetzung der Ausgrabungen am Hohle Fels und neue aurignacienzeitliche Werkzeuge aus Mammutelfenbein. *Archäologische Ausgrabungen in Baden-Württemberg* 56–59 (2019).](http://paperpile.com/b/k5bpNx/8hIB)

21. [Conard, N. J. & Uerpmann, H.-P. Die Ausgrabungen 1997 und 1998 im Hohle Fels bei Schelklingen, Alb-Donau-Kreis. *Archäologische Ausgrabungen in Baden-Württemberg* 47–52 (1998).](http://paperpile.com/b/k5bpNx/I9Aq)

22. [Bataille, G. & Conard, N. J. Blade and bladelet production at Hohle Fels Cave, AH IV in the Swabian Jura and its importance for characterizing the technological variability of the Aurignacian in Central Europe. *PLoS One* **13**, e0194097 (2018).](http://paperpile.com/b/k5bpNx/55Fo)

23. [Conard, N. J. & Bolus, M. Radiocarbon dating the late Middle Paleolithic and the Aurignacian of the Swabian Jura. *J. Hum. Evol.* **55**, 886–897 (2008).](http://paperpile.com/b/k5bpNx/w7Uo)

24. [Conard, N. J. & Bolus, M. Radiocarbon dating the appearance of modern humans and timing of cultural innovations in Europe: new results and new challenges. *J. Hum. Evol.* **44**, 331–371 (2003).](http://paperpile.com/b/k5bpNx/Si3f)

25. [Taller, A. & Conard, N. J. Transition or replacement? Radiocarbon dates from hohle fels cave (alb-donau-kreis/d) and the passage from aurignacian to gravettian. *Archaologisches Korrespondenzblatt* **49**, 165–181 (2019).](http://paperpile.com/b/k5bpNx/5nbU)

26. [Miller, C. E. *A Tale of Two Swabian Caves - Geoarchaeological Investigations at Hohle Fels and Geißenklösterle*. (Kerns Verlag Tübingen, 2015).](http://paperpile.com/b/k5bpNx/MOTS)

27. [Taller, A., Bolus, M. & Conard, N. J. The Magdalenian of Hohle Fels Cave and the Resettlement of the SWabian Jura after the LGM. 17 (2012).](http://paperpile.com/b/k5bpNx/tkGd)

28. [Barbieri, A. *et al.* Interpreting gaps: A geoarchaeological point of view on the Gravettian record of Ach and Lone valleys (Swabian Jura, SW Germany). *J. Archaeol. Sci.* **127**, 105335 (2021).](http://paperpile.com/b/k5bpNx/fPcK)

29. [Riehl, S., Marinova, E., Deckers, K., Malina, M. & Conard, N. J. Plant use and local vegetation patterns during the second half of the Late Pleistocene in southwestern Germany. *Archaeol. Anthropol. Sci.* **7**, 151–167 (2015).](http://paperpile.com/b/k5bpNx/DfAE)

30. [Wong, G. L., Starkovich, B. M., Drucker, D. G. & Conard, N. J. New perspectives on human subsistence during the Magdalenian in the Swabian Jura, Germany. *Archaeol. Anthropol. Sci.* **12**, 217 (2020).](http://paperpile.com/b/k5bpNx/N4D3)

31. [Jónsson, H., Ginolhac, A., Schubert, M., Johnson, P. L. F. & Orlando, L. mapDamage2.0: fast approximate Bayesian estimates of ancient DNA damage parameters. *Bioinformatics* **29**, 1682–1684 (2013).](http://paperpile.com/b/k5bpNx/UrSo)

32. [Rohland, N., Harney, E., Mallick, S., Nordenfelt, S. & Reich, D. Partial uracil-DNA-glycosylase treatment for screening of ancient DNA. *Philos. Trans. R. Soc. Lond. B Biol. Sci.* **370**, (2015).](http://paperpile.com/b/k5bpNx/SAxy)

33. [Reimer, P. J. *et al.* The IntCal20 Northern Hemisphere Radiocarbon Age Calibration Curve (0–55 cal kBP). *Radiocarbon* **62**, 725–757 (2020).](http://paperpile.com/b/k5bpNx/MyvQ)

34. [Ramsey, C. B. Bayesian Analysis of Radiocarbon Dates. *Radiocarbon* **51**, 337–360 (2009/ed).](http://paperpile.com/b/k5bpNx/6S2e)

**Supplementary Figures**

*
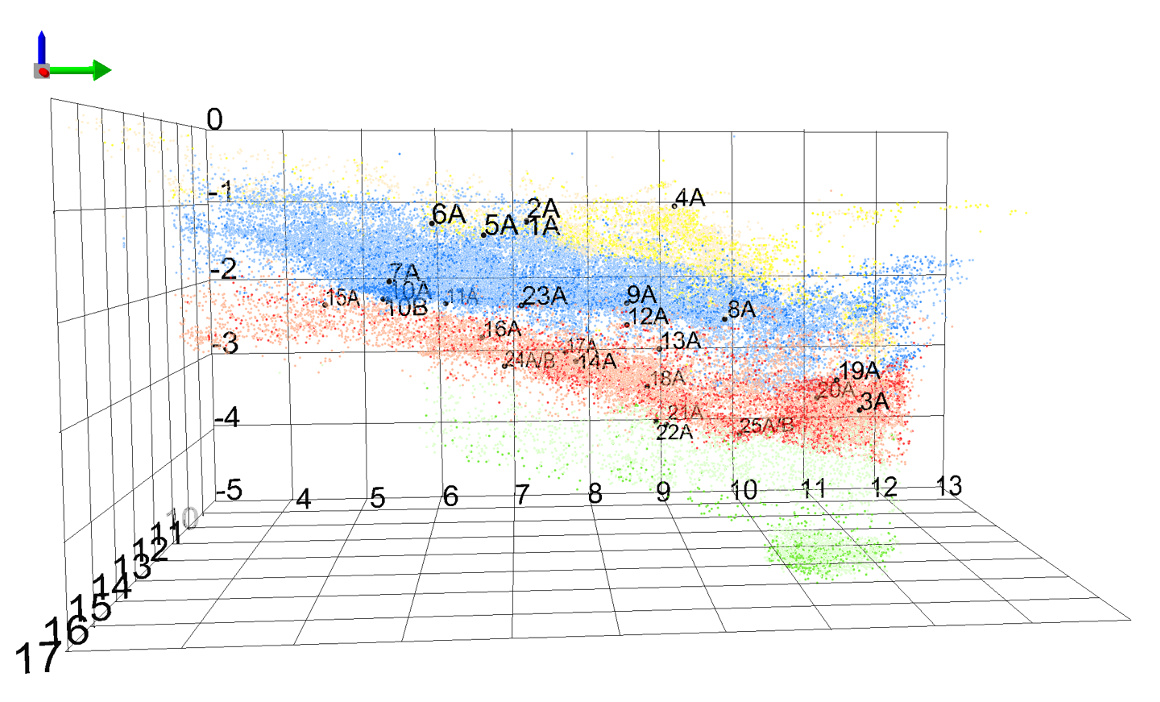
*

Figure S1: Selected ivory specimens amidst all finds from Hohle Fels. Yellow dots are finds associated with Magdalenian, Blue with Gravettian, Red with Aurignacian, and Green with Middle Paleolithic.


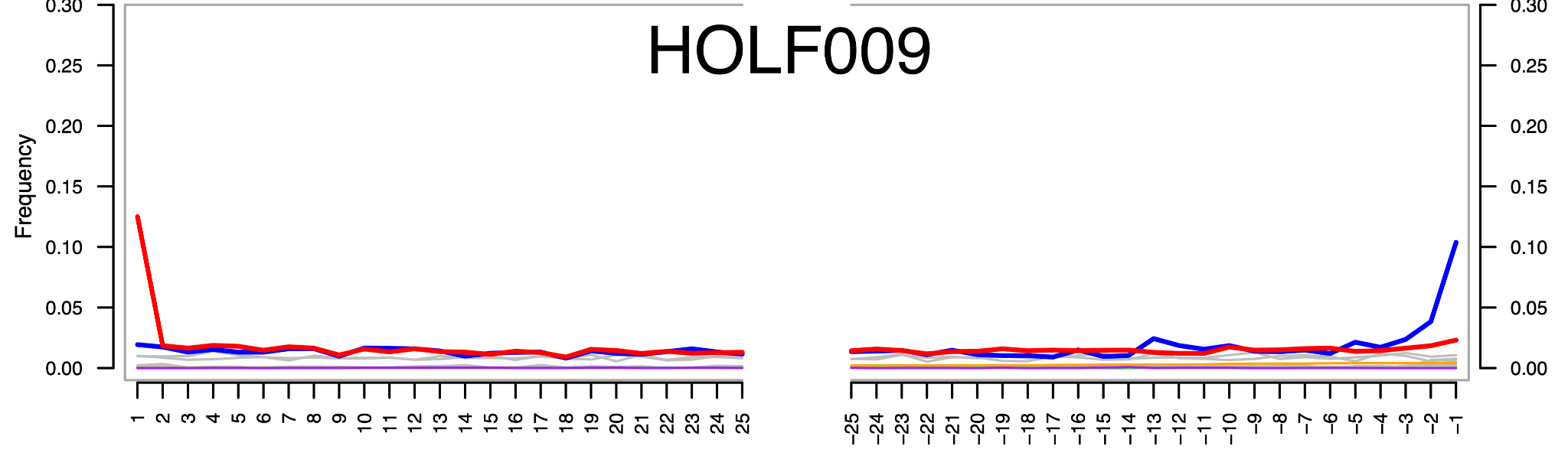

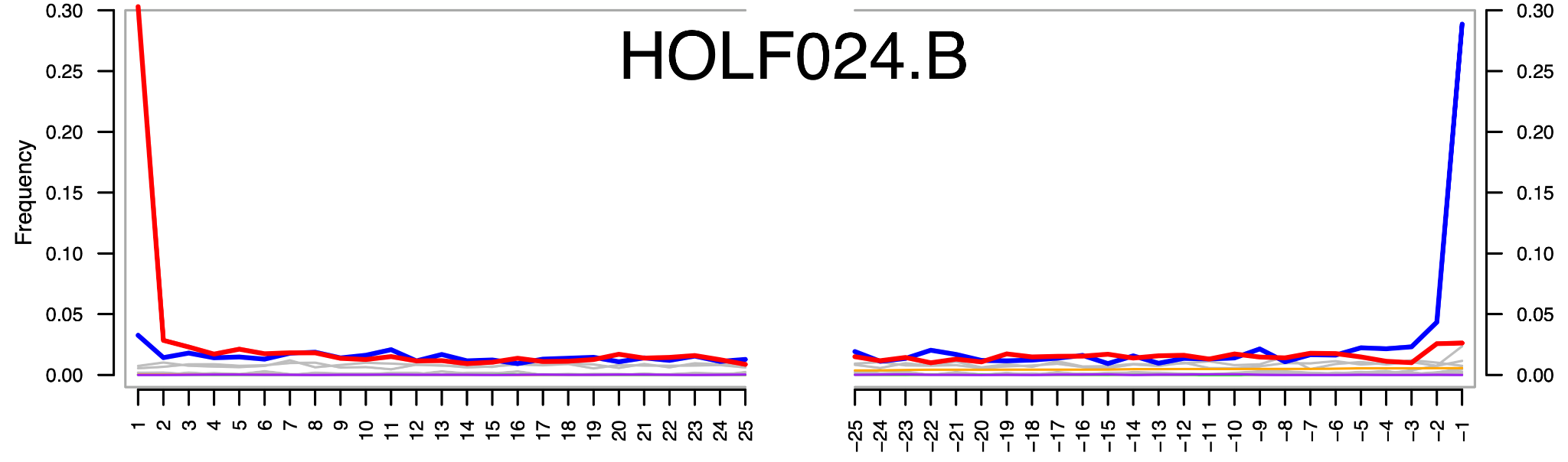

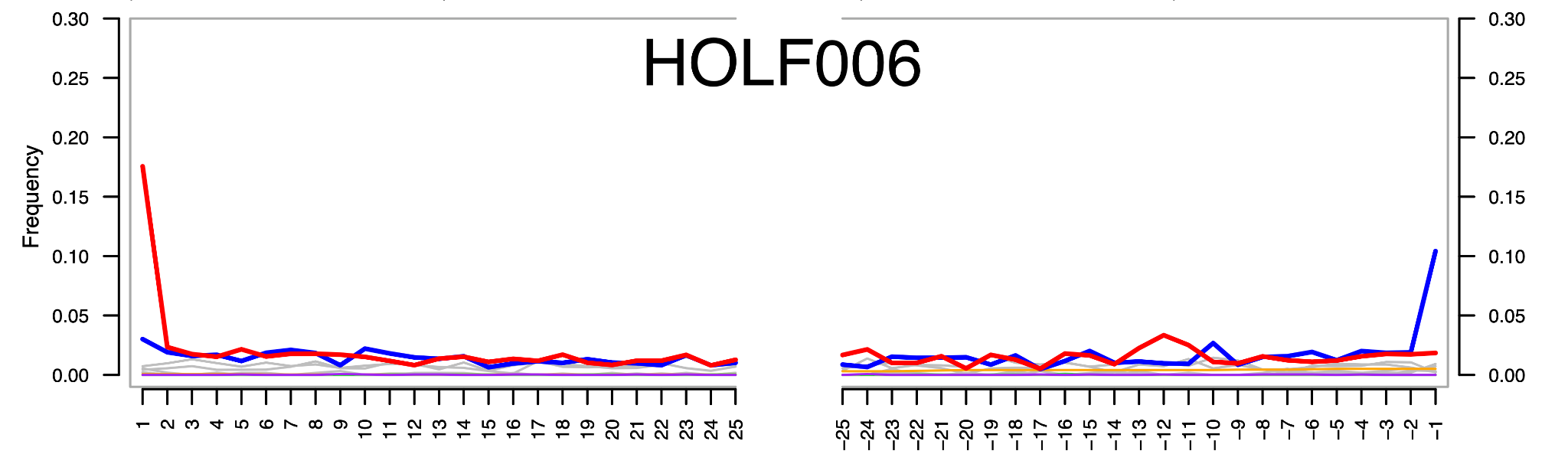

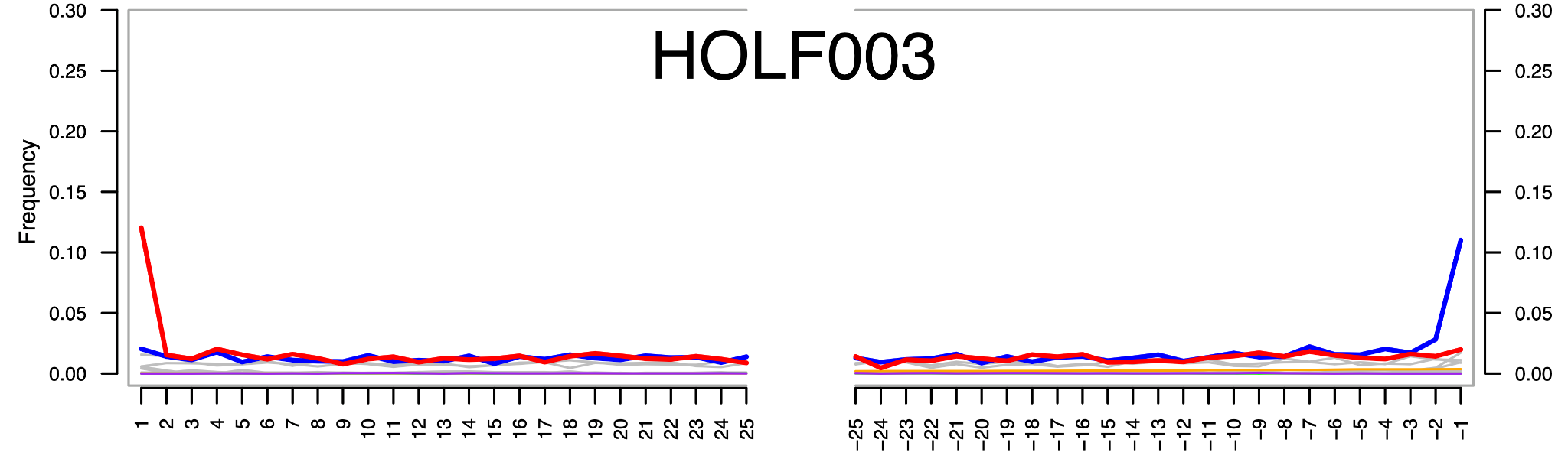

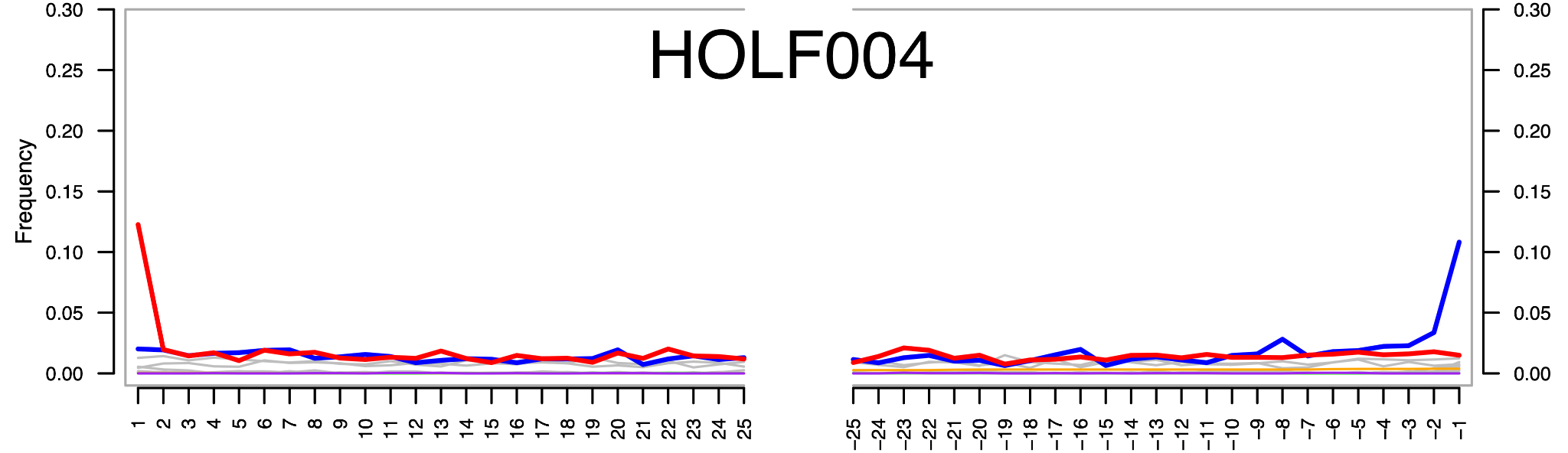

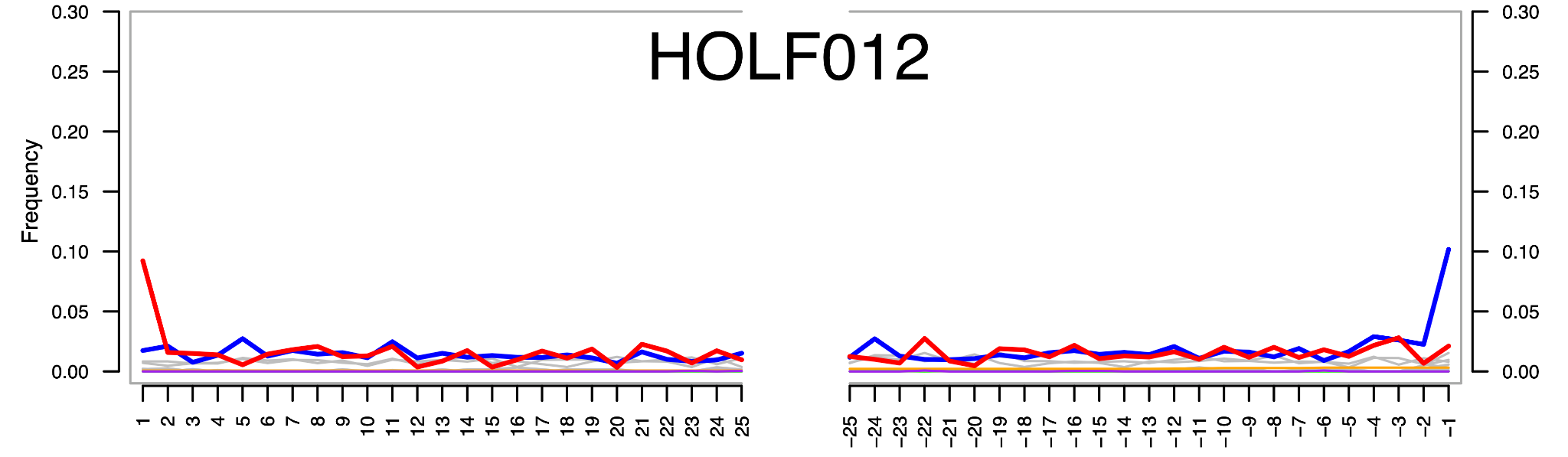

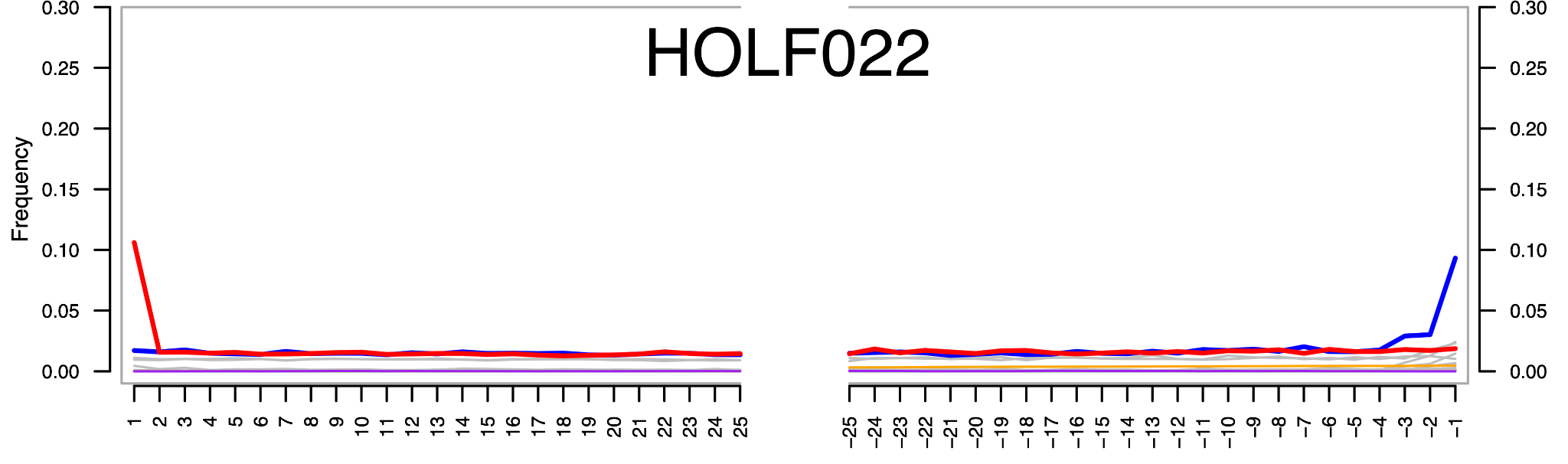

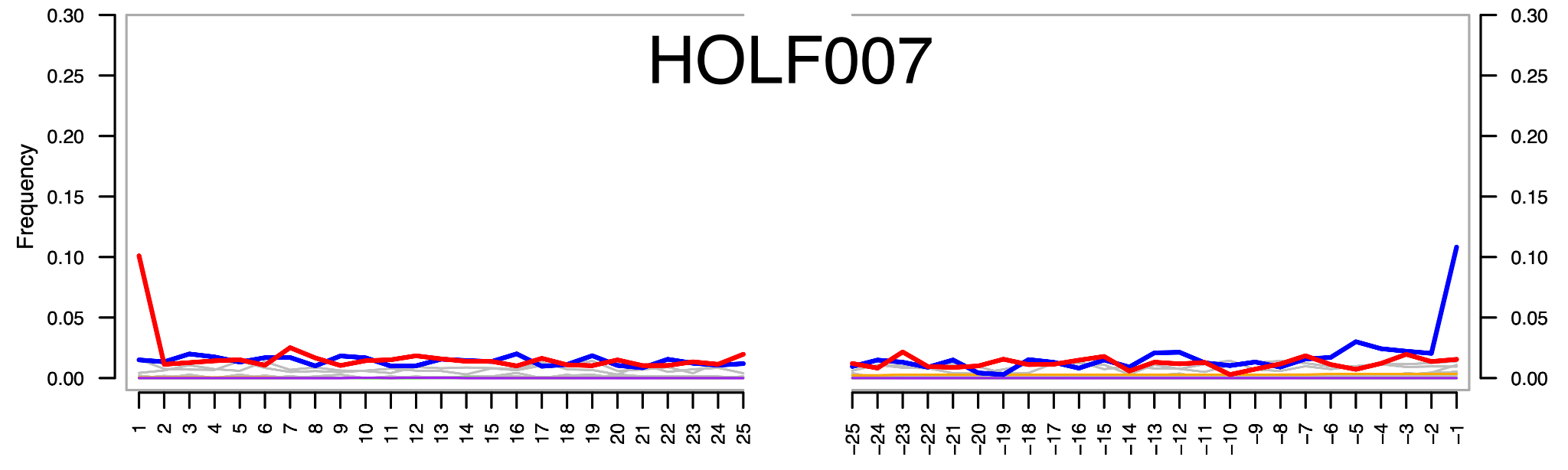

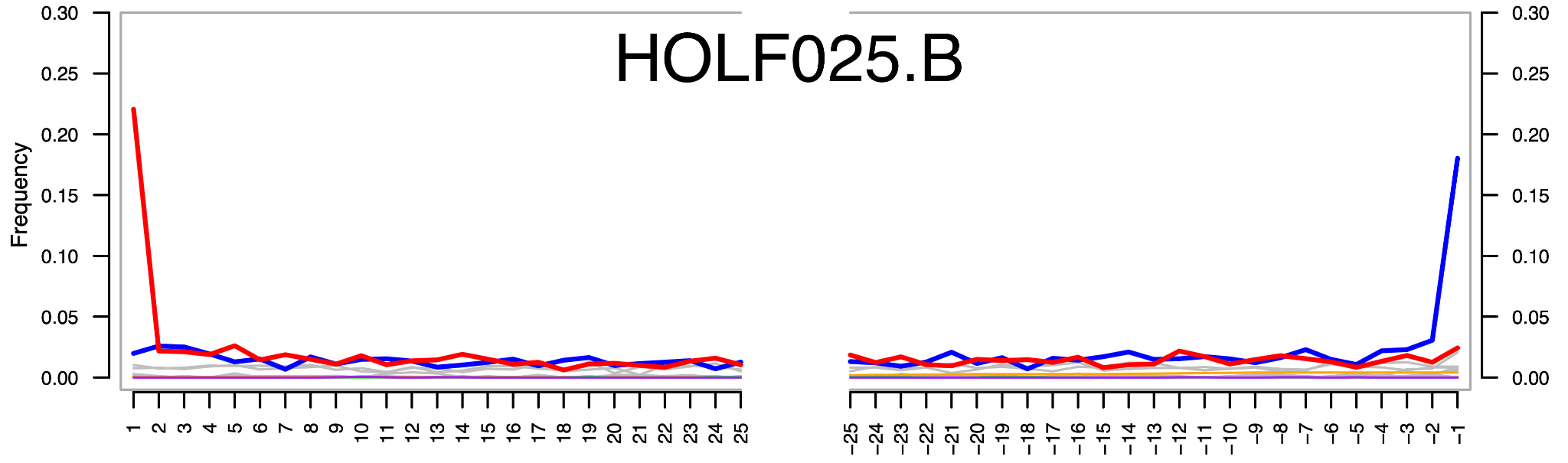

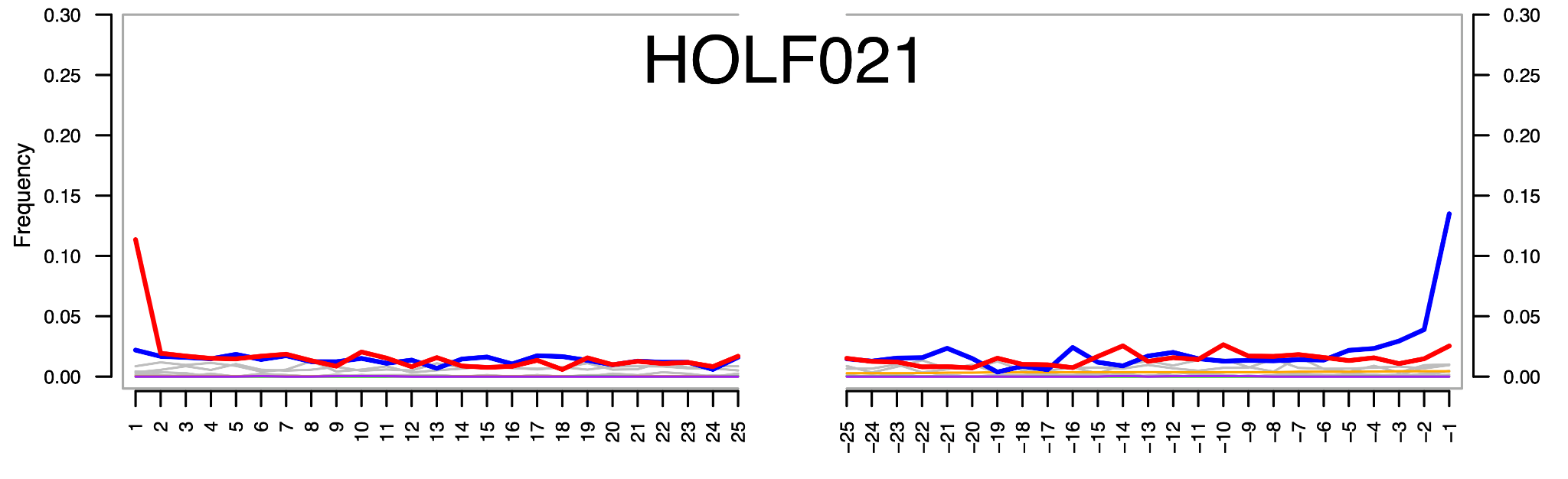

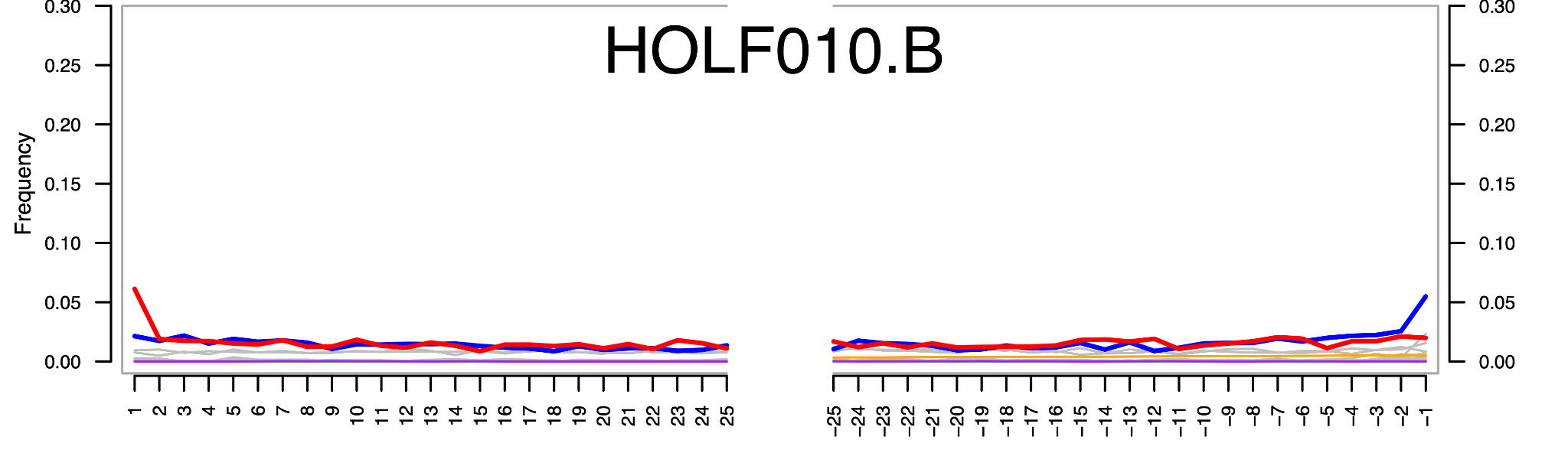

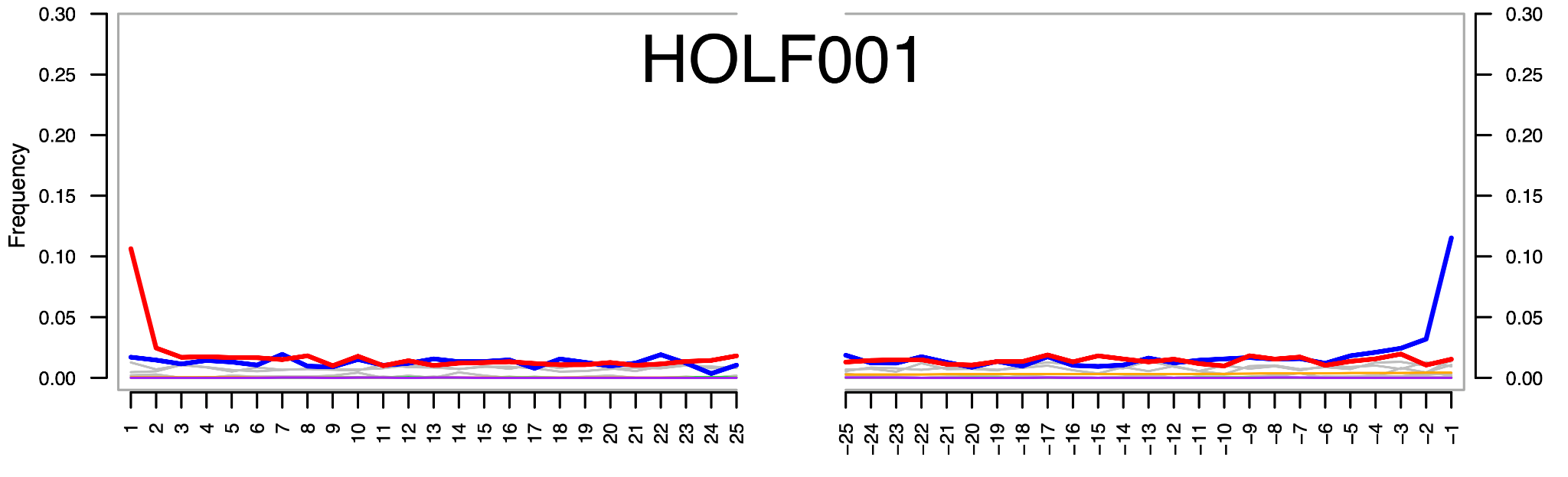


Figure S2: MapDamage plots of newly sequenced samples used in the mtDNA phylogeny [^31^](https://paperpile.com/c/k5bpNx/UrSo). All samples were partially UDG treated [^32^](https://paperpile.com/c/k5bpNx/SAxy).


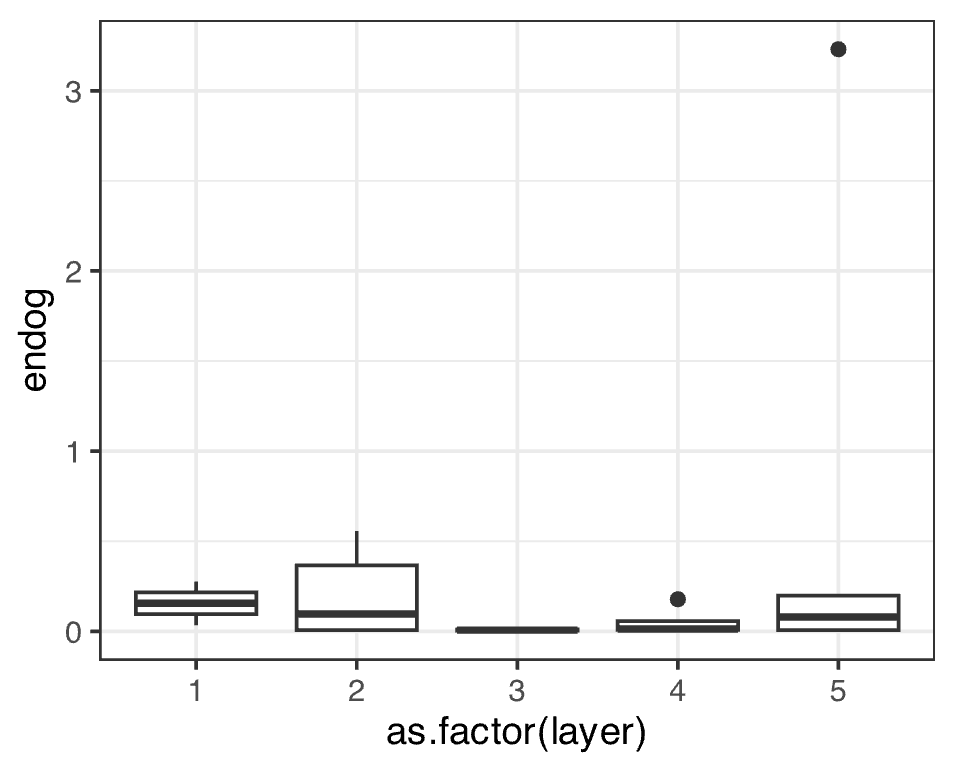


Figure S3: Endogenous DNA by Layer


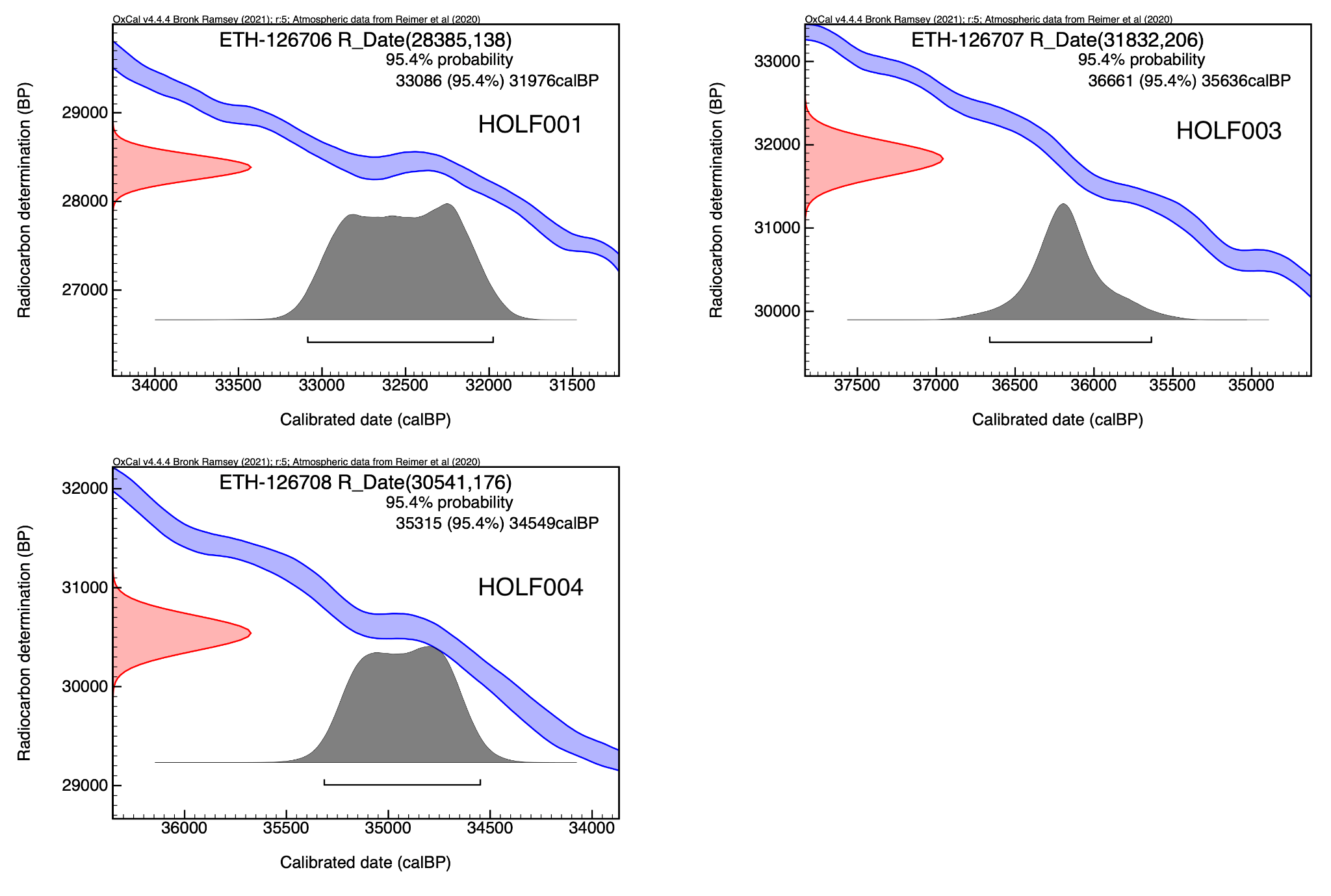


Figure S4: Radiocarbon dating calibration curves [^33^](https://paperpile.com/c/k5bpNx/MyvQ).


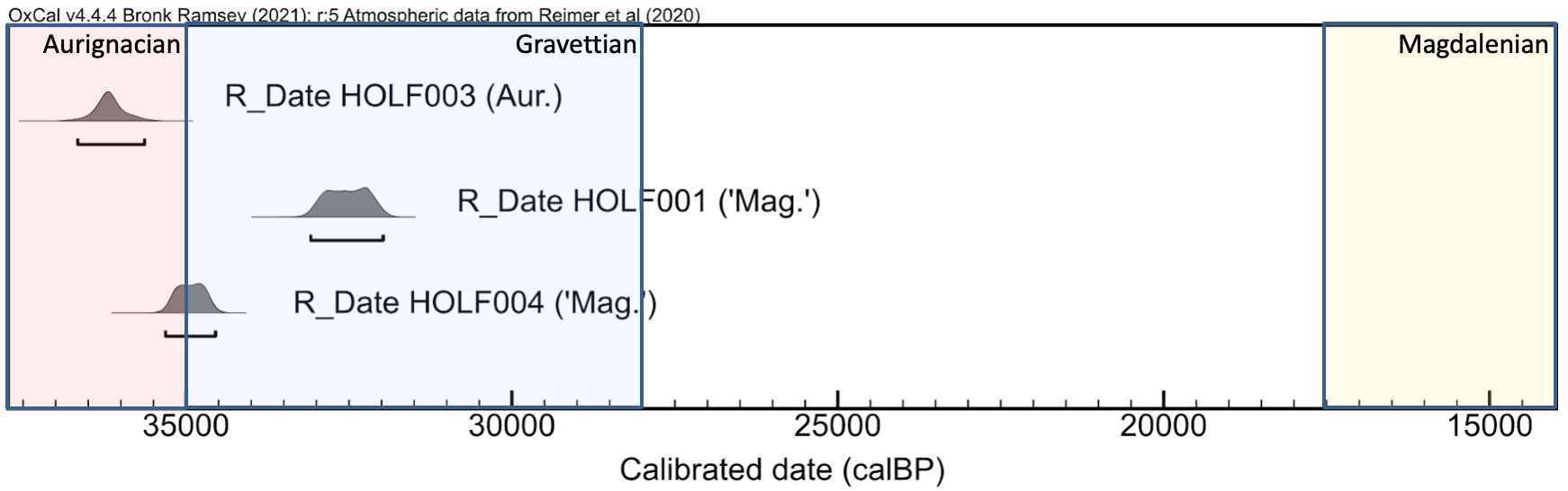


Figure S5: New radiocarbon dates plotted in OxCal [^34^](https://paperpile.com/c/k5bpNx/6S2e). HOLF001 and HOLF004 were found in layers associated with the Magdalenian in Hohle Fels but have radiocarbon dates consistent with the Gravettian culture.


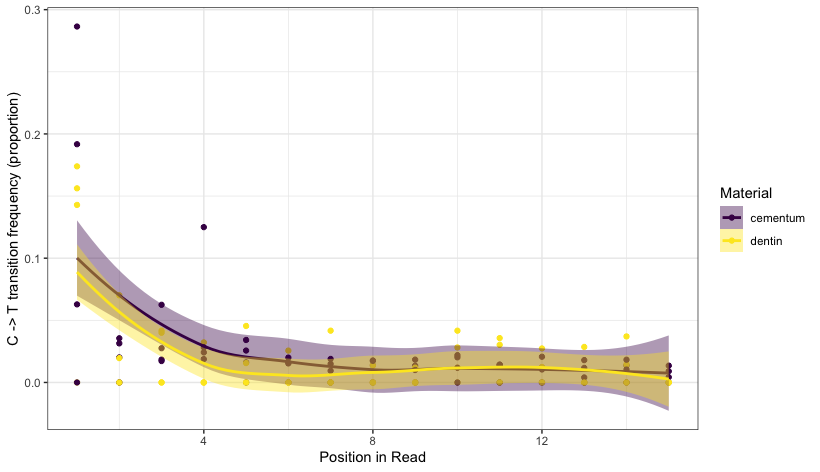


Figure S6: Damage patterns for cementum and dentin samples. Error ribbons display the 95% confidence interval of the fitted smoothing curve.

**
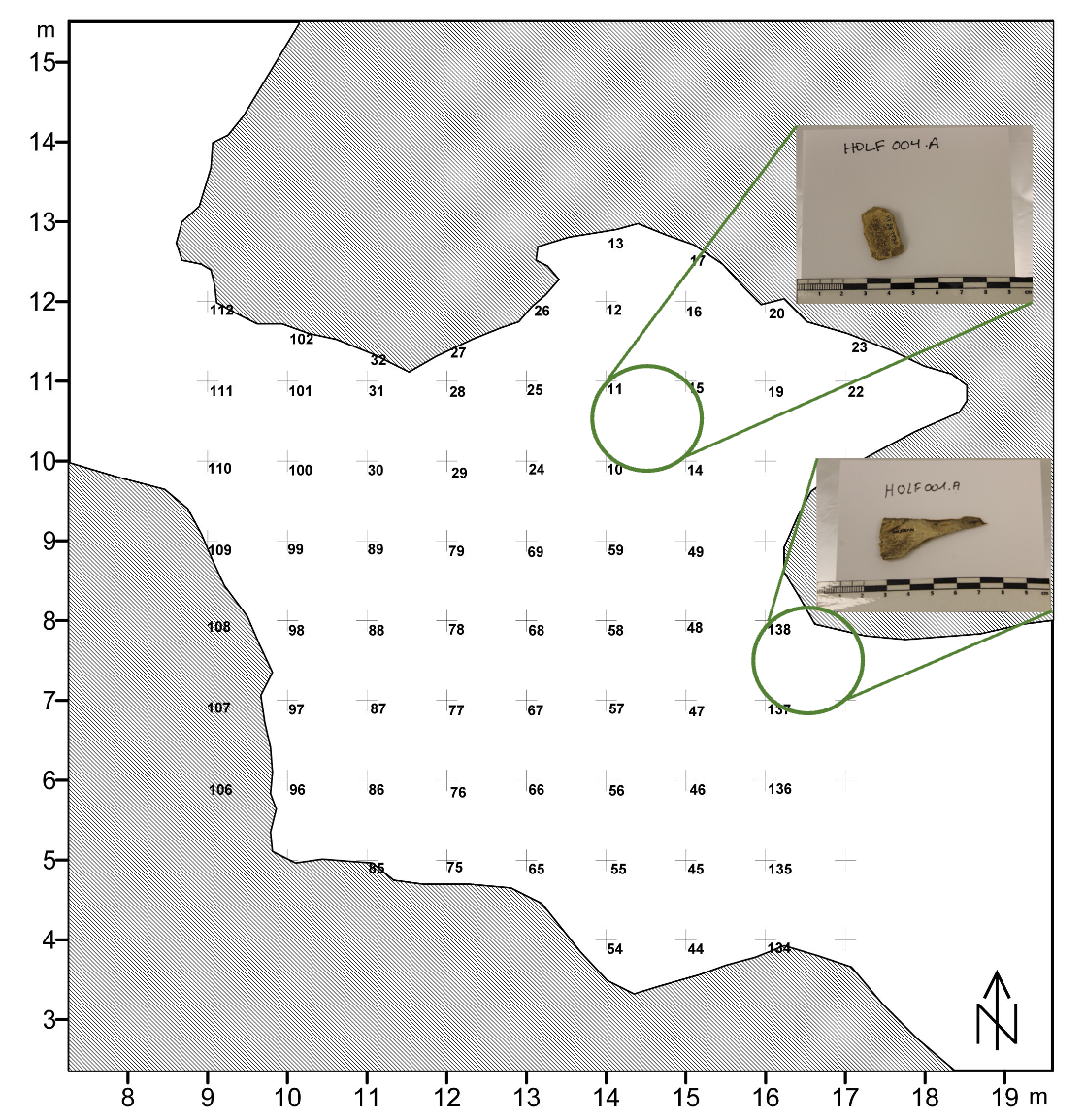
**

Figure S7: Archaeological quadrants for supposedly Magdalenian finds processed in this study. HOLF001 and HOLF002 share a quadrant, but only the photo for HOLF001 is shown as it was used in downstream analyses. The quadrants are consistent with the gullies described in [^28^](https://paperpile.com/c/k5bpNx/fPcK).
